# RED-ML: a novel, effective RNA editing detection method based on machine learning

**DOI:** 10.1093/gigascience/gix012

**Published:** 2017-03-02

**Authors:** Heng Xiong, Dongbing Liu, Qiye Li, Mengyue Lei, Liqin Xu, Liang Wu, Zongji Wang, Shancheng Ren, Wangsheng Li, Min Xia, Lihua Lu, Haorong Lu, Yong Hou, Shida Zhu, Xin Liu, Yinghao Sun, Jian Wang, Huanming Yang, Kui Wu, Xun Xu, Leo J. Lee

**Affiliations:** 1 BGI-Shenzhen, Shenzhen 518083, China; 2 China National GeneBank-Shenzhen, BGI-Shenzhen, Shenzhen 518083, China; 3 Department of Urology, Shanghai Changhai Hospital, Second Military Medical University, Shanghai 200433, China; 4 Department of Biology, University of Copenhagen, DK-2200 Copenhagen N, Denmark; 5 James D. Watson Institute of Genome Sciences, Hangzhou 310058, China; 6 Department of Electrical and Computer Engineering, Donnelly Centre for Cellular and Biomolecular Research, University of Toronto, Toronto, Ontario M5S 3G4, Canada

**Keywords:** RNA editing, A-to-I editing, RNA-seq, posttranscriptional modification, machine learning

## Abstract

With the advancement of second generation sequencing techniques, our ability to detect and quantify RNA editing on a global scale has been vastly improved. As a result, RNA editing is now being studied under a growing number of biological conditions so that its biochemical mechanisms and functional roles can be further understood. However, a major barrier that prevents RNA editing from being a routine RNA-seq analysis, similar to gene expression and splicing analysis, for example, is the lack of user-friendly and effective computational tools. Based on years of experience of analyzing RNA editing using diverse RNA-seq datasets, we have developed a software tool, RED-ML: RNA Editing Detection based on Machine learning (pronounced as “red ML”). The input to RED-ML can be as simple as a single BAM file, while it can also take advantage of matched genomic variant information when available. The output not only contains detected RNA editing sites, but also a confidence score to facilitate downstream filtering. We have carefully designed validation experiments and performed extensive comparison and analysis to show the efficiency and effectiveness of RED-ML under different conditions, and it can accurately detect novel RNA editing sites without relying on curated RNA editing databases. We have also made this tool freely available via GitHub <https://github.com/BGIRED/RED-ML>. We have developed a highly accurate, speedy and general-purpose tool for RNA editing detection using RNA-seq data. With the availability of RED-ML, it is now possible to conveniently make RNA editing a routine analysis of RNA-seq. We believe this can greatly benefit the RNA editing research community and has profound impact to accelerate our understanding of this intriguing posttranscriptional modification process.

## Introduction

RNA editing provides a dynamic and flexible means to alter the sequence of RNA transcripts during development and in a cell-type specific manner. Since discovered almost 30 years ago [1, 2], the biological importance of RNA editing, in particular adenosine to inosine (A-to-I) editing which is the most prevalent type in animals, has been well established [3–8]. Being a layer of posttranscriptional modification, it could increase the proteomic diversity of mRNA transcripts, affect transcript stability and localization, interact with other primary RNA processing steps such as splicing and polyadenylation, impact the biogenesis and functions of small RNAs such as microRNA and long noncoding RNA, and regulate gene expression. When misregulated, it contributes to various diseases [9, 10], including neurological disorders [11, 12] and cancer [13–16]. However, in spite of some well-studied examples, there is still much to be learned about the regulation and function of RNA editing in general.

In the last few years, large-scale, genome-wide analyses of RNA editing finally became feasible with the availability of high-throughput RNA sequencing [17, 18]. Even so, technical limitations and computational challenges have made this task difficult, especially at the beginning [19]. Several groups have since developed techniques to overcome many of the early difficulties with considerable success [20–24]. Nonetheless, the detection and quantification of RNA editing are still mostly restricted to a few specialized labs, partly due to the high demand of domain specific knowledge and skills to apply these methods effectively, as well as various usability issues of previous methods. A common theme of many previous RNA editing detection (RED) methods, including our own [17, 25], is to apply a series of carefully tuned filters to combat different types of errors affecting RED, such as sequencing artifacts, mapping errors, contamination from genomic variants, etc., in addition to the possible use of a second read alignment program [26]. While highly effective, these hard filters are difficult to adjust, tend to work well only under specific conditions, and cannot be easily modified to achieve different trade-offs between sensitivity and specificity.

Envisioning that deep, high-throughput RNA sequencing will keep acting as a driving force of RNA editing research, we have developed a fast, high performance, and user-friendly RED tool based on machine learning (ML) to better serve the community and advance the field. Our new tool RED-ML (RNA Editing Detection based on Machine Learning) can perform genome-wide RED based on human RNA-seq data alone, can take advantage of matching DNA-seq data if available, and integrates well with other common RNA-seq data analysis steps. By adopting ML principles [27], our new method can automatically and optimally combine different sources of information to detect RNA editing sites with adjustable confidence levels in a robust manner, and comes as a computationally efficient, all-in-one software package. To facilitate training and testing of our ML model, we have also carefully designed high-throughput RED validation experiments. In the remainder of this paper, we will first describe the design and components of our method, followed by comparisons and detailed analyses to verify its high performance, before concluding the paper with a discussion on further improvements and future directions.

## Methods

**Figure 1:**
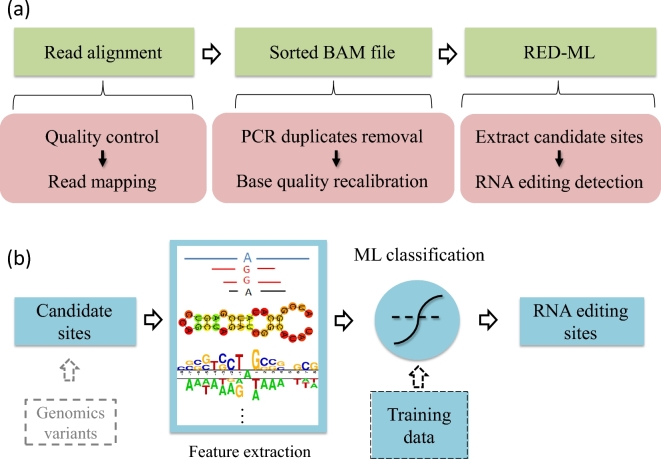
Flow charts of our RED-ML pipeline: (**a**) overview of the entire pipeline; (**b**) schematic of the ML component in RED-ML.

A flow chart of our RED pipeline using RED-ML is shown in Fig. 1a. The input to RED-ML is a sorted BAM file. Based on this sorted BAM file, RED-ML will extract candidate RNA editing sites and their corresponding features, with optional filtering if individual genotype information is available, then apply a logistic regression (LR) classifier to detect true RNA editing sites with an associated confidence score. Below we provide further details about the features used by RED-ML and the construction of the LR classifier.

### Features used by RED-ML

There are three broad classes of features used by RED-ML, based on insights obtained from previous hard filtering approaches, our own experience of tuning these filters, and current understanding of RNA editing mechanism. The first class is basic read features, including the number of supporting reads of a candidate site and the putative editing frequency. The second class of features is related to possible sequencing artifacts and misalignments, including mapping qualities of the supporting reads, the relative position of the candidate site in the mapped reads, indication of strand bias, whether the candidate site falls into simple repeat regions, etc. The third class is based on known properties of RNA editing, such as the editing type (whether it is A-to-I), whether the candidate site is in an Alu region, and its sequence context. Note that while the first two classes of features could be directly used in hard filtering, the third class cannot, since it is inappropriate to make hard decisions based on them, that is, they cannot be used as criteria to directly filter out non-RNA editing sites. However, they still provide valuable information to ML-based approaches where different sources of evidence can be combined to make soft decisions. In total, we extracted 28 features for every possible editing site, and full details of each feature are provided in Table S4.

### Validating RNA editing sties

To construct a classifier by supervised machine learning, it is imperative to have a high quality, adequate-sized training set on RNA editing. Unfortunately, the lack of a gold standard dataset is a well-known challenge in the field [19]. Here, we overcame this difficulty with a two-step strategy: first, we overlapped results of three previously developed RED methods on the same male Han Chinese individual RNA-seq and DNA-seq data [17], abbreviated as the YH dataset hereafter; second, we designed high-throughput experiments to validate RNA editing with high accuracy.

The three computational methods considered include the original one developed with the publication of the data by Peng et al. [17], a second method developed by a different lab shortly after by Ramaswami et al. [20], and an adapted and optimized version of RES-scanner [25] on the YH dataset (details in SM). Roughly speaking, the method by Peng et al. tends to be very accurate at the price of reduced sensitivity; the method by Ramaswami et al. substantially improved sensitivity but could be less accurate, while our own hard filters attempt to strike a balance between accuracy and sensitivity (Fig. S2 showing the Venn diagram, details in SM). Overall, due to the many differences among the three methods and independent validation experiments carried out in the first two, it is very likely that the overlap of these three, which is shown in Fig. S2, consists of genuine RNA editing sites.

To further validate these predicted RNA editing sites, we carried out high-throughput Ion Proton sequencing [28] (details in SM) using the same YH sample. Although both Ion Proton sequencing and Illumima Hiseq are referred to as second generation sequencing platforms, they differ in many key aspects, including the underlying chemistry, base calling method, as well as read alignment strategies. We took advantage of these differences to perform independent, high-throughput validation of the RNA-editing sites detected by Hiseq. In contrast, other validation methods that have been used in the literature, such as Sanger sequencing and mass spectrometry (MS), are of low-throughout and limited sensitivity, and not able to generate a dataset of reasonable size and diversity that can be used to train a ML classifier. To confirm the effectiveness of our high-throughput Ion Proton validation method, we checked whether the sites predicted by Peng et al. could be confidently detected. As shown in Fig. S1, most of the predicted sites with adequate Ion Proton sequencing coverage are detected (details in SM), with increasing validation rate as the sequencing coverage increases. Since sites predicted by Peng at al. tend to be highly accurate, this further justifies the soundness of our Ion Proton validation approach. Based on the trend shown in Fig. S1, we picked a coverage threshold of 20 when evaluating the performance of RED-ML in the Results section.

### Building a ML classifier

To build a high quality classifier based on ML principles, we carefully constructed the positive and negative training sets as follows. The positive set contains the overlap of three hard-filtering based RED methods (2960 sites) that are further validated by Ion Proton sequencing with a minimum coverage of 15, which results in 1334 sites (the slightly reduced coverage threshold is to obtain a large enough positive set). In addition, we also selected sites detected by both Peng et al. and Ramaswami et al., but not our own method, that are validated by Ion Proton sequencing (Fig. S2). This gives us an additional 141 validated RNA editing sites and results in a total of 1475 data points in the positive set. To construct the negative set, we first selected seven highly informative features used by our hard filtering method that are also shared by RED-ML, and randomly sampled 150 sites each that failed the corresponding hard filtering criterion, which results in 1050 data points. We also sampled 300 sites that were aligned by TopHat2 but filtered out by BWA, and not validated by Ion Proton sequencing. We further randomly sampled 1200 SNPs from dbSNP 138 so that the classifier can be trained to distinguish between typical SNPs and RNA editing sites. Finally, we added those RNA editing sites that are detected by only one or two of the three methods but not validated by Ion Proton sequencing even when the coverage is adequate (20x or more), which results in an additional 375 data points. This gives us 2925 negative samples overall and a total of 4400 data points in the training set (full details in SM).

We tried several popular ML techniques to build classifiers for RNA editing detection and settled on logistic regression due to its simplicity, efficiency of implementation, and relatively good performance (further discussions later). The LR classifier was trained and tested using the scikit-learn Python package (version 0.17.1), with a slightly higher weight (2.0) given to positive points to minimize the *F*_0.5_ score, which is defined as ${F_{0.5}} = ( {1 + {{0.5}^2}} )\ \bullet \frac{{{\rm{precision}} \bullet {\rm{recall}}}}{{( {{{0.5}^2} \bullet {\rm{precision}}} ) + {\rm{recall}}}}$. Five-fold cross validation and grid search were carried out to pick between L1 and L2 regularization and an appropriate regularization coefficient to avoid overfitting. A LR classifier with weak L2 regularization was selected as the final architecture. The final LR classifier was trained on the full set of 4401 data points using the best hyper-parameters picked by cross validation and grid search.

## Results

**Figure 2:**
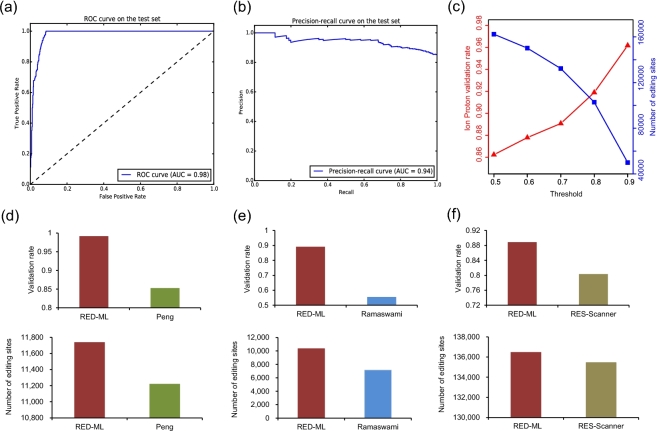
Evaluating RED-ML on the YH dataset. (**a** and **b**) Receiver operating characteristic and precision-recall curves on the test set when building the LR classifier. Both curves were plotted to show a more comprehensive picture of RED-ML performance on this biased dataset, where the number of negative examples is about twice of the positive ones, and they are obtained by varying the detection threshold in small steps. (**c**) The effect of varying the detection threshold: the Ion Proton validation rate increases monotonically as more stringent classification thresholds are chosen. (**d** and **f**) Adjusting the detection threshold to compare RED-ML with the methods of Peng et al, Ramaswami et al. and RES-scanner: the thresholds used are 0.96, 0.5, and 0.68, respectively.

The set of 4400 data points just described would be very challenging for hard filtering based approaches. To test the performance of our ML based approach, we randomly partitioned these data points into training (80%) and test (the remaining 20%) sets. Performance on test data, which is not used when training the model, is shown in Fig. 2a and b, where an area under curve of 0.98 for the receiver operating characteristic curve and an area under curve of 0.94 for the precision-recall curve were obtained, demonstrating the good performance of our LR classifier on this task. A key advantage of our ML based method is that it also outputs a confidence score of detection, which could be interpreted as the probability of a candidate site being a true RNA editing site. Therefore, this score provides a turning knob to adjust between sensitivity and specificity to suit different research goals, which is missing in hard filtering based approaches. As a test, we have applied our trained LR classifier on the full YH dataset and adjusted this threshold between the default 0.5 and the highly confident 0.9, and the Ion Proton validation rate increases monotonically as expected (Fig. 2c).

We further took advantage of such an ability to do pair-wise comparison with the other three methods used in building our model. For the method of Peng et al. and RES-scanner, we adjusted the threshold of RED-ML to roughly match the total number of detected RNA editing sites and compared the validation rates by Ion Proton sequencing. For the method of Ramaswami et al., we adjusted the threshold to match the number of detected RNA editing sites in non-Alu regions only, since Ramaswami et al. applied a very loose filter in the Alu region and included many low frequency sites that are not able to be detected by RED-ML (more details in SM). These results are shown in Fig. 2d, e and f, where RED-ML clearly outperforms the other three methods by detecting slightly more RNA editing sites while achieving higher Ion Proton validation rates at the same time. For example, when detecting ∼140 000 RNA editing sites similar to RES-scanner (with a threshold of 0.68), the validation rate of RED-ML is 0.88 while RES-scanner is 0.82. When using the default threshold of 0.5, the validation rate of RED-ML only dropped slightly to 0.86, still higher than that of RES-scanner, but it can detect ∼27 000 more RNA editing sites (Table S5.1).

**Figure 3:**
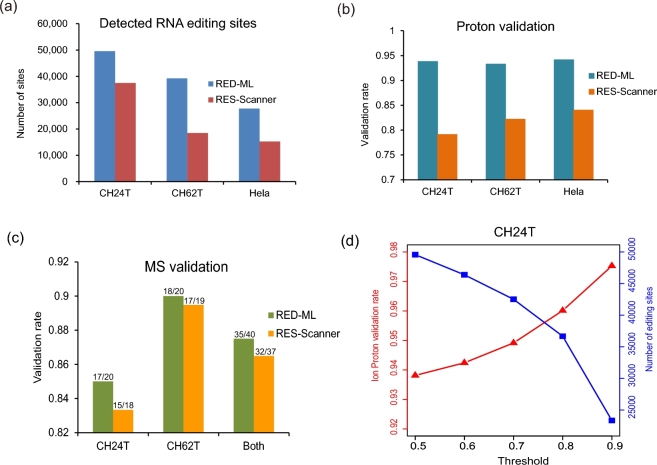
Evaluating RED-ML on two prostate tumor samples (CH24T and CH62T) and a Hela sample: (**a**) number of detected RNA editing sites and (**b**) Ion Proton validate rates by RED-ML (using the default detection threshold of 0.5) and RES-scanner in the three samples; (**c**) MS validation of some RNA editing sites detected by RED-ML and RES-scanner in CH24T and CH62T; (**d**) the effect of varying the detection threshold in CH24T.

It should be emphasized that evaluating RED-ML on the YH dataset is not truly unbiased, since a very small portion of the YH dataset has been used in training our model. Moreover, other methods have been more or less tuned on the YH dataset as well. Most importantly, however, is that a critical goal of adopting ML principles for RED is to build a tool that can generalize well, that is, by learning the intrinsic, underlying characteristics of RNA editing, it can reach high performance beyond a specific dataset, experimental setup or tissue type, etc. To fully test the real-world performance of RED-ML, we carried out independent RNA-seq experiments on two prostate tumor samples (CH24T and CH62T) and a HeLa sample to detect RNA editing with RED-ML, and further performed Ion Proton validation experiments on these samples. RED-ML detected ∼30 000–50 000 RNA editing sites using the default threshold of 0.5 (Fig. 3a, with full details in Tables S5.1 and S11) and achieved Ion Proton validation rates of 0.9 or higher in these three samples (Fig. 3b). We also applied RES-scanner as a high performance baseline to compare against, which has been demonstrated to be superior among existing RED methods [25]. Once again, RED-ML substantially outperforms RES-scanner on these three datasets (Fig. 3a and b), by detecting more RNA editing sites and simultaneously achieving higher validation rates. This clearly demonstrates the advantage of our new ML based approach, which can generalize well beyond the data used to train the model. We have also performed MS validation experiments on some detected sites in the prostate tumor samples, randomly selected across a wide range of RNA editing levels (15–90%) with a slight bias towards sites in non-Alu regions (Table S6) and achieved an overall validation rate of 87.5% (35/40, Fig. 3c). As before, the detection threshold can be further adjusted to detect fewer but more confident sites, and it achieved even higher validation rates (Fig. 3d).

**Figure 4:**
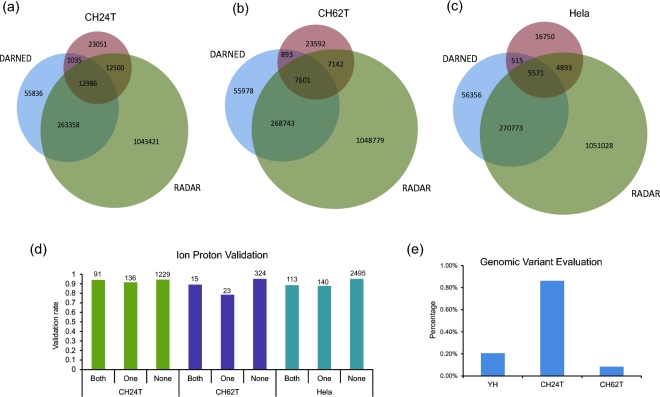
Further analysis of the RED-ML detected sites. (**a**–**c**) The overlap of detected sites with two curated RNA editing databases (DARNED and RADAR) in CH24T, CH62T, and Hela samples were shown as Venn diagrams. (**d**) Ion Proton validation rates for different classes of sites (defined in the main text) in the three samples. The number of validated sites in each class is also indicated on the top of each bar. (**e**) The percentage of genomic variants in detected RNA editing sites as quantified by matching DNA sequencing data.

RED-ML did not use information from existing RNA editing databases when detecting editing sites, which enables it to detect novel, sample-specific sites. This is a valuable asset in many applications, especially disease studies. To investigate whether it suffers from lower accuracy by not using curated databases, we carried out the following analysis. We first checked the overlap of RED-ML detected sites in CH24T, CH62T and Hela samples with those in two curated RNA editing databases (DARNED and RADAR) and plotted the results as Venn diagrams (Fig. 4a, b and c). Significant portions of RED-ML detected sites are in neither of the existing databases (46.5%, 60.1%, and 60.4% for CH24T, CH62T, and Hela samples, respectively), probably because these are not normal tissues. We then partitioned the detected RNA editing sites into three categories: (1) both: existed in both DARNED and RADAR; (2) one: existed in only one of DARNED and RADAR but not both; (3) none: existed in none of the two databases, and checked the validation rates of these three categories across three samples. As shown in Fig. 4d, there are no significant differences on the validation rates among the categories in all three samples, which demonstrate that RED-ML performed quite consistently independent of existing RNA editing databases. To study the effect of genomic variants on RED, we compared the sites detected by RED-ML (without using genomic variant information) with the genomic variants detected by DNA sequencing on the same sample (Fig. 4e). Even in the highly challenging tumor samples, where there exist both somatic SNVs and SNPs, the percentage of genomic variants in RED-ML detected RNA editing sites is quite low (no more than 1%), which confirms the high specificity of RED-ML in detecting RNA editing sites.

Running the RED-ML pipeline from a sorted BAM file only takes a single command, and it runs quite fast for typical RNA-seq experiments, usually no more than an overnight job. For example, using a single thread on a Linux machine with a quad-core AMD Opteron 2.4-GHz processor, it takes 5–8 hours for CH24T, CH62T, and Hela samples and ∼16 hours for the much larger YH dataset (Table S7.1), with no more than 5GB RAM usage. Most of the computation time was on variant pileup, while the ML step is extremely fast (∼10 minutes for all samples). Compared to our previously published RES-scanner, the improvement on speed is very substantial, achieving ∼6x–10x speedup (Table S7.2). This is mainly due to the removal of a time-consuming realignment step by BLAT, as well as some optimization of variant pileup.

## Discussions

In conclusion, a highly effective and widely applicable RED tool based on ML has been developed. We have also adopted careful software design to make this tool easy to use and it comes as an all-in-one software package. In addition, by adopting ML principles in building our model, further improvement can be easily made when improved knowledge of RNA editing becomes available. For example, when more accurate, large-scale RNA editing validation results are available, we can retrain our model with a better training set. When more characteristics of the RNA editing mechanism are discovered, we can design more features to reflect our improved knowledge.

One limitation of RED-ML is that it only detects RNA editing sites with relatively high editing levels. The lowest level in our training set is 0.1, and RED-ML rarely detects sites with levels lower than 0.1 in reality. This limitation is mostly by design, since we aim to detect functional RNA editing sites, which are unlikely to be of very low frequency, and it also helps to reduce the impact of sequencing errors and artifacts. However, if the accuracy in sequencing experiments and alignment tools could be substantially improved, such a limitation can be readily lifted when building our model. The speed of RED-ML can also be further improved if multithreading is supported in the variant pileup and feature extraction stage, and we plan to do so in the future. Meanwhile, a user could process the BAM files of each chromosome in parallel to speed up the pipeline.

Although RED-ML can accept BAM files produced by different alignment tools, the current version has been specifically optimized for BWA and TopHat2 due to the construction of model, and we find that the choice of alignment tools and the associated parameters could have a large impact on RED. To help users with proper alignment strategies, we have detailed some recommendations in the SM. We have also tested some alignment tools other than those used in building our model. For example, when we tried the BAM file produced by STAR [29] on the CH24T dataset, we detected many RNA editing sites but with low validation rate (∼0.34, details in SM). When we tried the BAM file produced by HISAT2 [30], which could be considered as the successor of TopHat2, the result is much better (validation rate ∼0.85, A-to-I ∼0.93, details in SM), probably due to its similarity to TopHat2. Since designing accurate RNA-seq alignment strategies, especially in the context of SNP and RNA editing detection, is still an open research problem [24], we plan to incorporate more popular alignment tools when building future versions of RED-ML.

The current version of RED-ML is designed for human RED since we used various features specific to human RNA editing as well as human data when building our ML model. With the increased RNA editing data available in other species as well as the growing interest of studying them, we could build future versions to support more species, as our previous method RES-scanner did. As a test, we have run RED-ML on ant BAM files from RNA-seq data in Li et al [31] by disabling all human related features. The result does not seem to be good qualitatively. For example, the percentage of A-to-I editing is only ∼60% (details in SM), and it shows that more work needs to be done to make RED-ML work well on other species.

A simple ML technique, namely LR, has been adopted in the current version of RED-ML. We also tried other methods, including decision trees, random forests, and SVMs, but the gain in performance by more sophisticated techniques is very minor (data not shown). As a result, LR was picked since it runs very fast and can be easily incorporated into our existing RNA-seq pipeline. However, when the need is warrantied, more sophisticated ML techniques, including deep learning [32], could be applied. ML may play a particularly large role when the accumulation of data and knowledge on RNA editing reaches such a stage that computational models of RNA editing could be assembled to simulate the process, such as what has been successfully accomplished for RNA splicing [33], or even building joint models with other RNA processing steps, and we believe this is a promising direction of future RNA editing research.

## Availability and requirements

Project name: RED-ML

Project home page: https://github.com/BGIRED/RED-ML

Operating system(s): Linux_x86_64

Programming language: Perl & C++

Other requirements: SAMtools package and the following Perl modules: FindBin, Getopt::Long, File::Basename.

License: GNU General Public License version 3.0 (GPLv3)

Any restrictions to use by non-academics: None

## Availability of Supporting Data

Data further supporting this work can be found in the GigaScience repository, GigaDB [34]. More information can also be found in the project homepage [35].

**FigureS1.pdf**

**FigureS2.pdf**

**FigureS3.pdf**

**FigureS4.pdf**

**FigureS5.pdf**

**FigureS6.pdf**

**FigureS7.pdf**

**FigureS8.pdf**

**TableS1-10.xlsx**

**TableS11.xlsx**

## Competing interests

The authors declare that they have no competing interests.

## Author contributions

LJL and DL designed the study; HX, DL, LJL, QL, and CW contributed to the software programming and pipeline construction; SR, LW, and LX prepared the samples; LW, LX, WL, MX, LL, and HL performed the experiment; HX and ML analyzed the data; YH, SZ, XL, YS, JW, HY, KW, and XX supervised the project; LJL, HX, and DL wrote the manuscript; all authors read and approved the final manuscript.

## Additional file

Additional file: Fig. S1: validation of RNA editing sites identified by Peng et al. [1] binned according to Ion Proton sequencing coverage depth. Validation rate increases with increased coverage, and it roughly saturates at 20x depth.

Additional file: Fig. S2: overlapping detected RNA editing sites among three methods (RES-Scanner, Peng et al. [1], Ramaswami et al [5]).

Additional file: Fig. S3: the significance of features for RNA editing detection by RED-ML. The features were sorted orderly according to its weights.

Additional file: Fig. S4: comparison of RED-ML and RES-Scanner (Tophat2). To ensure a fair comparison, we have compared the RED-ML and RES-Scanner based on same alignment file (Tophat2, because of RES-Scanner also accepted Tophat2 alignment file [6]) (**a**) The numbers of editing sites identified by RED-ML are larger than those of RES-Scanner (Tophat2) in CH24T, CH62T, and Hela. (**b**) The Ion Proton validation rates of editing sites identified by RED-ML are higher than those by RES-Scanner (Tophat2) in CH24T, CH62T, and Hela. RED-ML has a greater advantage since TopHat2 has been used in constructing our LR classifier while RES-Scanner has only been optimized for BWA.

Additional file: Fig. S5: the number of RNA editing sites and Ion Proton validation rate under different thresholds. With the threshold increasing, the numbers of detected editing sites decrease and the Ion Proton validation rates increase. **a**, **b**, and **c** show the CH62T and Hela samples, respectively.

Additional file: Fig. S6: comparison with known RNA editing database. (**a**–**c**) The overlap of detected sites with two curated RNA editing databases (DARNED and RADAR) in CH24T, CH62T, and Hela samples were shown as Venn diagrams. Significant portions of RED-ML detected sites are in neither of the existing databases (37.5%, 36.5%, and 59.2% for CH24T, CH62T, and Hela samples, respectively), probably because these are not normal tissues.

Additional file: Fig. S7: Ion Proton validation rates for different classes of sites (defined in the main text) in the three samples. The number of Ion Proton validated sites in each class is also indicated on the top of each bar. There are no significant differences on the validation rates among the categories in all three samples, which demonstrate that RED-ML performed quite consistently independent of existing RNA editing databases.

Additional file: Fig. S8: SNP evaluation. The percentage of genomic variants in detected RNA editing sites as quantified by matching DNA sequencing data. And the percentage of genomic variants in RED-ML detected RNA editing sites is quite low (no more than 2%).

Additional file: Table S4. The definitions of 28 selected features.

Additional file: Table S11. The RNA editing sites of CH24T, CH62T, Hela and YH detected by RED-ML.

## Supplementary Material

GIGA-D-16-00159_Original_Submission.pdf

Reviewer_1_Report_(Original_Submission).pdf

Reviewer_2_Report_(Original_Submission).pdf

FigureS1.pdf

FigureS2.pdf

FigureS3.pdf

FigureS4.pdf

FigureS5.pdf

FigureS6.pdf

FigureS7.pdf

FigureS8.pdf

TableS1-10.xlsx

TableS11.xlsx
