# Supplementary material for: RED-ML: a novel, effective RNA editing detection method based on machine learning
Source: Gigascience. 2017 Mar 2;6(5):1–8. doi: 10.1093/gigascience/gix012 (PMC5467039; doi:10.1093/gigascience/gix012)
Supplement: GIGA-D-16-00159_Original_Submission.pdf [file gix012_GIGA-D-16-00159_Original_Submission.pdf]

Manuscript Click here to download Manuscript RED-ML with DOI.docx

RED-ML: a novel, effective RNA editing detection method based on machine learning

Heng Xiong[1,2*], Dongbing Liu[1,2*], Qiye Li[1,2], Mengyue Lei[1,2], Liqin Xu[1,2], Liang Wu[1,2], Zongji Wang[1,2], Shancheng Ren[3], Wangsheng Li[1,2], Min Xia[1,2], Lihua Lu[1,2], Haorong Lu[1,2], Yong Hou[1,2,4], Shida Zhu[1,2,4], Xin Liu[1,2], Yinghao Sun[3], Jian Wang[1,5], Huanming Yang[1,5], Kui Wu[1,2,4], Xun Xu[1,2#], and Leo J Lee[1,6#]

[1]BGI-Shenzhen, Shenzhen 518083, China
[2]China National GeneBank-Shenzhen, BGI-Shenzhen, Shenzhen 518083, China
[3]Department of Urology, Shanghai Changhai Hospital, Second Military Medical University, Shanghai 200433, China
[4]Department of Biology, University of Copenhagen, DK-2200 Copenhagen N, Denmark
[5]James D. Watson Institute of Genome Sciences, Hangzhou 310058, China
[6]Department of Electrical and Computer Engineering, Donnelly Centre for Cellular and Biomolecular Research, University of Toronto, Toronto, Ontario M5S 3G4, Canada
[*]Equal contributors
[#]Correspondence: ljlee@psi.toronto.edu, xuxun@genomics.cn;

Abstract

Background: With the advancement of second generation sequencing techniques, our ability to detect and quantify RNA editing on a global scale has been vastly improved. As a result, RNA editing is now being studied under a growing number of biological conditions so that its biochemical mechanisms and functional roles can be further understood. However, a major barrier that prevents RNA editing from being a routine RNA-seq analysis, similar to gene expression and splicing analysis for example, is the lack of user-friendly and effective computational tools.

Findings: Based on years of experience of analyzing RNA editing using diverse RNA-seq datasets, we have developed a software tool RED-ML: RNA Editing Detection based on Machine learning (pronounced as "red ML"). The input to RED-ML can be as simple as a single BAM file, while it can also take advantage of matched genomic variant information when available. The output not only contains detected RNA editing sites, but also a confidence score to facilitate downstream filtering. We have carefully designed validation experiments and performed extensive comparison and analysis to show the efficiency and effectiveness of RED-ML under different conditions, and it can accurately detect novel RNA editing sites without relying on curated RNA editing databases. We have also made this tool freely available via GitHub <https://github.com/BGIRED/RED-ML>.

Conclusions: We have developed a highly accurate, speedy and general-purpose tool for RNA editing detection using RNA-seq data. With the availability of RED-ML, it is now possible to conveniently make RNA editing a routine analysis of RNA-seq. We believe this can greatly benefit the RNA editing research community and has profound impact to accelerate our understanding of this intriguing post-transcriptional modification process.

Keywords: RNA editing, A-to-I editing, RNA-seq, post-transcriptional modification, machine learning

Introduction

RNA editing provides a dynamic and flexible means to alter the sequence of RNA transcripts during development and in a cell-type specific manner. Since discovered almost 30 years ago [1, 2], the biological importance of RNA editing, in particular adenosine to inosine (A-to-I) editing which is the most prevalent type in animals, has been well established [3-8]. Being a layer of post-transcriptional modification, it could increase the proteomic diversity of mRNA transcripts, affect transcript stability and localization, interact with other primary RNA processing steps such as splicing and polyadenylation, impact the biogenesis and functions of small RNAs such as microRNA (miRNA) and long noncoding RNA (lncRNA) and regulate gene expression. When mis-regulated, it contributes to various diseases [9, 10], including neurological disorders [11, 12] and cancer [13-16]. However, in spite of some well-studied examples, there is still much to be learned about the regulation and function of RNA editing in general.

In the last few years, large-scale, genome-wide analyses of RNA editing finally became feasible with the availability of high throughput RNA sequencing [17, 18]. Even so, technical limitations and computational challenges have made this task difficult, especially at the beginning [19]. Several groups have since developed techniques to overcome many of the early difficulties with considerable success [20-24]. Nonetheless, the detection and quantification of RNA editing are still mostly restricted to a few specialized labs, partly due to the high demand of domain specific knowledge and skills to apply these methods effectively, as well as various usability issues of previous methods. A common theme of many previous RNA editing detection (RED) methods, including our own [17, 25], is to apply a series of carefully tuned filters to combat different types of errors affecting RED, such as sequencing artifacts, mapping errors, contamination from genomic variants etc, in addition to the possible use of a second read alignment program [26]. While highly effective, these hard filters are difficult to adjust, tend to work well only under specific conditions, and cannot be easily modified to achieve different trade-offs between sensitivity and specificity.

Envisioning that deep, high-throughput RNA sequencing will keep acting as a driving force of RNA editing research, we have developed a fast, high performance and user-friendly RED tool based on machine learning (ML) to better serve the community and advance the field. Our new tool RED-ML (RNA Editing Detection based on Machine Learning) can perform genome-wide RED based on human RNA-seq data alone, can take advantage of matching DNA-seq data if available, and integrates well with other common RNA-seq data analysis steps. By adopting ML principles [27], our new method can automatically and optimally combine different sources of information to detect RNA editing sites with adjustable confidence levels in a robust manner, and comes as a computationally efficient, all-in-one software package. To facilitate training and testing of our ML model, we have also carefully designed high-throughput RED validation experiments. In the remainder of this paper, we will first describe the design and components of our method, followed by comparisons and detailed analyses to verify its

high performance, before concluding the paper with a discussion on further improvements and future directions.

Methods

A flow chart of our RED pipeline using RED-ML is shown in Fig. 1a. The input to RED-ML is a sorted BAM file. Based on this sorted BAM file, RED-ML will extract candidate RNA editing sites and their corresponding features, with optional filtering if individual genotype information is available, then apply a logistic regression (LR) classifier to detect true RNA editing sites with an associated confidence score. Below we provide further details about the features used by RED-ML and the construction of the LR classifier.

Features used by RED-ML

There are three broad classes of features used by RED-ML, based on insights obtained from previous hard filtering approaches, our own experience of tuning these filters, and current understanding of RNA editing mechanism. The first class is basic read features, including the number of supporting reads of a candidate site and the putative editing frequency. The second class of features is related to possible sequencing artifacts and misalignments, including mapping qualities of the supporting reads, the relative position of the candidate site in the mapped reads, indication of strand bias, whether the candidate site falls into simple repeat regions etc. The third class is based on known properties of RNA editing, such as the editing type (whether it is A-to-I), whether the candidate site is in an Alu region and its sequence context. Note that while the first two classes of features could be directly used in hard filtering, the third class cannot since it is inappropriate to make hard decisions based on them, i.e., they cannot be used as criteria to directly filter out non-RNA editing sites. However, they still provide valuable information to ML based approaches where different sources of evidence can be combined to make soft decisions. In total, we extracted 28 features for every possible editing site, and full details of each feature are provided in Table S4.

Validating RNA editing sties

To construct a classifier by supervised machine learning, it is imperative to have a high quality, adequate-sized training set on RNA editing. Unfortunately, the lack of a gold standard dataset is a well-known challenge in the field [19]. Here, we overcame this difficulty with a two-step strategy: first, we overlapped results of three previously developed RED methods on the same male Han Chinese individual RNA-seq and DNA-seq data [17], abbreviated as the YH dataset hereafter; second, we designed high-throughput experiments to validate RNA editing with high accuracy.

The three computational methods considered include the original one developed with the publication of the data by Peng et al [17], a second method developed by a different lab shortly after by Ramaswami et al [20], and an adapted and optimized version of RES-

scanner [25] on the YH dataset (details in SM). Roughly speaking, the method by Peng et al tends to be very accurate at the price of reduced sensitivity; the method by Ramaswami et al substantially improved sensitivity but could be less accurate, while our own hard filters attempt to strike a balance between accuracy and sensitivity (Fig. S2 showing the Venn diagram, details in SM). Overall, due to the many differences among the three methods and independent validation experiments carried out in the first two, it is very likely that the overlap of these three, which is shown in Fig. S2, consists of genuine RNA editing sites.

To further validate these predicted RNA editing sites, we carried out high-throughput Ion Proton sequencing[28] (details in SM) using the same YH sample. Although both Ion Proton sequencing and Illumima Hiseq are referred to as second generation sequencing platforms, they differ in many key aspects, including the underlying chemistry, base calling method as well as read alignment strategies. We took advantage of these differences to perform independent, high-throughput validation of the RNA-editing sites detected by Hiseq. In contrast, other validation methods that have been used in the literature, such as Sanger sequencing and mass spectrometry (MS), are of low-throughout and limited sensitivity, and not able to generate a dataset of reasonable size and diversity that can be used to train a ML classifier. To confirm the effectiveness of our high-throughput Ion Proton validation method, we checked whether the sites predicted by Peng et al could be confidently detected. As shown in Fig. S1, most of the predicted sites with adequate Ion Proton sequencing coverage are detected (details in SM), with increasing validation rate as the sequencing coverage increases. Since sites predicted by Peng at al tend to be highly accurate, this further justifies the soundness of our Ion Proton validation approach. Based on the trend shown in Fig. S1, we picked a coverage threshold of 20 when evaluating the performance of RED-ML in the Results section.

Building a ML classifier

In order to build a high quality classifier based on ML principles, we carefully constructed the positive and negative training sets as follows. The positive set contains the overlap of three hard-filtering based RED methods (2,960 sites) that are further validated by Ion Proton sequencing with a minimum coverage of 15, which results in 1,334 sites (the slightly reduced coverage threshold is to obtain a large enough positive set). In addition, we also selected sites detected by both Peng et al and Ramaswami et al, but not our own method, that are validated by Ion Proton sequencing (Fig. S2). This gives us an additional 141 validated RNA editing sites and results in a total of 1,475 data points in the positive set. To construct the negative set, we first selected seven highly informative features used by our hard filtering method that are also shared by RED-ML, and randomly sampled 150 sites each that failed the corresponding hard filtering criterion, which results in 1,050 data points. We also sampled 300 sites that were aligned by TopHat2 but filtered out by BWA, and not validated by Ion Proton sequencing. We further randomly sampled 1,200 SNPs from dbSNP 138 so that the classifier can be trained to distinguish between typical SNPs and RNA editing sites. Finally, we added those RNA editing sites that are detected by only one or two of the three methods but not validated by Ion Proton sequencing even when the coverage is adequate (20x or more),

which results in an additional 375 data points. This gives us 2,925 negative samples overall and a total of 4,400 data points in the training set (full details in SM).

We tried several popular ML techniques to build classifiers for RNA editing detection and settled on logistic regression due to its simplicity, efficiency of implementation and relatively good performance (further discussions later). The LR classifier was trained and tested using the scikit-learn Python package (version 0.17.1), with a slightly higher weight (2.0) given to positive points to minimize the $F_{0.5}$ score, which is defined as $F_{0.5} = (1 + 0.5^2) \cdot \frac{\text{precision} \cdot \text{recall}}{(0.5^2 \cdot \text{precision}) + \text{recall}}$. Five fold cross validation and grid search were carried out to pick between L1 and L2 regularization and an appropriate regularization coefficient to avoid overfitting. A LR classifier with weak L2 regularization was selected as the final architecture. The final LR classifier was trained on the full set of 4,401 data points using the best hyper-parameters picked by cross validation and grid search.

Results

The set of 4,400 data points just described would be very challenging for hard filtering based approaches. To test the performance of our ML based approach, we randomly partitioned these data points into training (80%) and test (the remaining 20%) sets. Performance on test data, which is not used when training the model, is shown in Fig. 2a & 2b, where an area under curve (AUC) of 0.98 for the receiver operating characteristic (ROC) curve and an AUC of 0.94 for the precision-recall curve were obtained, demonstrating the good performance of our LR classifier on this task. A key advantage of our ML based method is that it also outputs a confidence score of detection, which could be interpreted as the probability of a candidate site being a true RNA editing site. Therefore, this score provides a turning knob to adjust between sensitivity and specificity to suit different research goals, which is missing in hard filtering based approaches. As a test, we have applied our trained LR classifier on the full YH dataset and adjusted this threshold between the default 0.5 and the highly confident 0.9, and the Ion Proton validation rate increases monotonically as expected (Fig. 2c).

We further took advantage of such an ability to do pair-wise comparison with the other three methods used in building our model. For the method of Peng et al and RES-scanner, we adjusted the threshold of RED-ML to roughly match the total number of detected RNA editing sites and compared the validation rates by Ion Proton sequencing. For the method of Ramaswami et al, we adjusted the threshold to match the number of detected RNA editing sites in non Alu regions only, since Ramaswami et al applied a very loose filter in the Alu region and included many low frequency sites that are not able to be detected by RED-ML (more details in SM). These results are shown in Fig. 2d, 2e & 2f, where RED-ML clearly outperforms the other three methods by detecting slightly more RNA editing sites while achieving higher Ion Proton validation rates at the same time. For example, when detecting ~140,000 RNA editing sites similar to RES-scanner (with a threshold of 0.68), the validation rate of RED-ML is 0.88 while RES-scanner is 0.82. When using the default threshold of 0.5, the validation rate of RED-ML only dropped

slightly to 0.86, still higher than that of RES-scanner, but it can detect ~27,000 more RNA editing sites (Table S5.1).

It should be emphasized that evaluating RED-ML on the YH dataset is not truly unbiased, since a very small portion of the YH dataset has been used in training our model. Moreover, other methods have been more or less tuned on the YH dataset as well. Most importantly, however, is that a critical goal of adopting ML principles for RED is to build a tool that can generalize well, i.e., by learning the intrinsic, underlying characteristics of RNA editing, it can reach high performance beyond a specific dataset, experimental setup or tissue type etc. To fully test the real world performance of RED-ML, we carried out independent RNA-seq experiments on two prostate tumor samples (CH24T and CH62T) and a HeLa sample to detect RNA editing with RED-ML, and further performed Ion Proton validation experiments on these samples. RED-ML detected ~30,000-50,000 RNA editing sites using the default threshold of 0.5 (Fig. 3a, with full details in Tables S5.1 and S11) and achieved Ion Proton validation rates of 0.9 or higher in these three samples (Fig. 3b). We also applied RES-scanner as a high performance baseline to compare against, which has been demonstrated to be superior among existing RED methods [25]. Once again, RED-ML substantially outperforms RES-scanner on these three datasets (Fig. 3 a&b), by detecting more RNA editing sites and simultaneously achieving higher validation rates. This clearly demonstrates the advantage of our new ML based approach, which can generalize well beyond the data used to train the model. We have also performed mass spectrometry (MS) validation experiments on some detected sites in the prostate tumor samples, randomly selected across a wide range of RNA editing levels (15%-90%) with a slight bias towards sites in non-Alu regions (Table S6) and achieved an overall validation rate of 87.5% (35/40, Fig. 3c). As before, the detection threshold can be further adjusted to detect fewer but more confident sites, and it achieved even higher validation rates (Fig. 3d).

RED-ML didn't use information from existing RNA editing databases when detecting editing sites, which enables it to detect novel, sample-specific sites. This is a valuable asset in many applications, especially disease studies. To investigate whether it suffers from lower accuracy by not using curated databases, we carried out the following analysis. We first checked the overlap of RED-ML detected sites in CH24T, CH62T and Hela samples with those in two curated RNA editing databases (DARNED and RADAR) and plotted the results as Venn diagrams (Fig. 4 a, b & c). Significant portions of RED-ML detected sites are in neither of the existing databases (46.5%, 60.1% and 60.4% for CH24T, CH62T and Hela samples respectively), probably because these are not normal tissues. We then partitioned the detected RNA editing sites into three categories: (1) both: existed in both DARNED and RADAR; (2) one: existed in only one of DARNED and RADAR but not both; (3) none: existed in none of the two databases, and checked the validation rates of these three categories across three samples. As shown in Fig. 4d, there are no significant differences on the validation rates among the categories in all three samples, which demonstrate that RED-ML performed quite consistently independent of existing RNA editing databases. In order to study the effect of genomic variants on RED, we compared the sites detected by RED-ML (without using genomic variant information) with the genomic variants detected by DNA sequencing on the same sample (Fig. 4e).

Even in the highly challenging tumor samples, where there exist both somatic SNVs and SNPs, the percentage of genomic variants in RED-ML detected RNA editing sites is quite low (no more than 1%), which confirms the high specificity of RED-ML in detecting RNA editing sites.

Running the RED-ML pipeline from a sorted BAM file only takes a single command, and it runs quite fast for typical RNA-seq experiments, usually no more than an overnight job. For example, using a single thread on a Linux machine with a quad-core AMD Opteron 2.4GHz processor, it takes 5-8 hours for CH24T, CH62T and Hela samples and ~16 hours for the much larger YH dataset (Table S7.1), with no more than 5GB RAM usage. Most of the computation time was on variant pileup, while the ML step is extremely fast (~10 minutes for all samples). Comparing to our previously published RES-scanner, the improvement on speed is very substantial, achieving ~6x-10x speedup (Table S7.2). This is mainly due to the removal of a time-consuming realignment step by BLAT, as well as some optimization of variant pileup.

Discussions

In conclusion, a highly effective and widely applicable RED tool based on ML has been developed. We have also adopted careful software design to make this tool easy to use and it comes as an all-in-one software package. In addition, by adopting ML principles in building our model, further improvement can be easily made when improved knowledge of RNA editing becomes available. For example, when more accurate, large-scale RNA editing validation results are available, we can retrain our model with a better training set. When more characteristics of the RNA editing mechanism are discovered, we can design more features to reflect our improved knowledge.

One limitation of RED-ML is that it only detects RNA editing sites with relatively high editing levels. The lowest level in our training set is 0.1, and RED-ML rarely detects sites with levels lower than 0.1 in reality. This limitation is mostly by design since we aim to detect functional RNA editing sites, which are unlikely to be of very low frequency, and it also helps to reduce the impact of sequencing errors and artifacts. However, if the accuracy in sequencing experiments and alignment tools could be substantially improved, such a limitation can be readily lifted when building our model. The speed of RED-ML can also be further improved if multithreading is supported in the variant pileup and feature extraction stage, and we plan to do so in the future. Meanwhile, a user could process the BAM files of each chromosome in parallel to speed up the pipeline.

Although RED-ML can accept BAM files produced by different alignment tools, the current version has been specifically optimized for BWA and TopHat2 due to the construction of model, and we find that the choice of alignment tools and the associated parameters could have a large impact on RED. To help users with proper alignment strategies, we have detailed some recommendations in the SM. We have also tested some alignment tools other than those used in building our model. For example, when we tried the BAM file produced by STAR [29] on the CH24T dataset, we detected many RNA

editing sites but with low validation rate (~0.34, details in SM). When we tried the BAM file produced by HISAT2 [30], which could be considered as the successor of TopHat2, the result is much better (validation rate ~0.85, A-to-I ~0.93, details in SM), probably due to its similarity to TopHat2. Since designing accurate RNA-seq alignment strategies, especially in the context of SNP and RNA editing detection, is still an open research problem [24], we plan to incorporate more popular alignment tools when building future versions of RED-ML.

The current version of RED-ML is designed for human RED since we used various features specific to human RNA editing as well as human data when building our ML model. With the increased RNA editing data available in other species as well as the growing interest of studying them, we could build future versions to support more species, as our previous method RES-scanner did. As a test, we have run RED-ML on ant BAM files from RNA-seq data in Li et al [31] by disabling all human related features. The result doesn't seem to be good qualitatively. For example, the percentage of A-to-I editing is only ~60% (details in SM), and it shows that more work needs to be done to make RED-ML work well on other species.

A simple ML technique, namely logistic regression (LR), has been adopted in the current version of RED-ML. We also tried other methods, including decision trees, random forests and SVMs, but the gain in performance by more sophisticated techniques is very minor (data not shown). As a result, LR was picked since it runs very fast and can be easily incorporated into our existing RNA-seq pipeline. However, when the need is warrantied, more sophisticated ML techniques, including deep learning [32], could be applied. ML may play a particularly large role when the accumulation of data and knowledge on RNA editing reaches such a stage that computational models of RNA editing could be assembled to simulate the process, such as what has been successfully accomplished for RNA splicing [33], or even building joint models with other RNA processing steps, and we believe this is a promising direction of future RNA editing research.

Availability and requirements
Project name: RED-ML
Project home page: https://github.com/BGIRED/RED-ML
Operating system(s): Linux_x86_64
Programming language: Perl & C++
Other requirements: SAMtools package and the following Perl modules: FindBin, Getopt::Long, File::Basename.
License: GNU General Public License version 3.0 (GPLv3)
Any restrictions to use by non-academics: None

Availability of Supporting Data
Data further supporting this work can be found in the GigaScience repository, GigaDB [34]. More information can also be found in the project homepage [35].

Competing interests
The authors declare that they have no competing interests.

Authors' contributions
LJL and DL designed the study; HX, DL, LJL, QL and CW contributed to the software programming and pipeline construction; SR, LW and LX prepared the samples; LW, LX, WL, MX, LL and HL performed the experiment; HX and ML analyzed the data; YH, SZ, XL, YS, JW, HY, KW and XX supervised the project; LJL, HX and DL wrote the manuscript; All authors read and approved the final manuscript.

Acknowledgements
This project was supported by the Shenzhen Peacock Plan (NO.KQTD20150330171505310). We are indebted to colleagues of Beijing Genomics Institute who contributed to this work but were not included in the author list. We also thank Dr. Laurie Goodman and Dr. Scott Edmunds for their valuable suggestions.

Reference
1. Bass BL, Weintraub H: **A developmentally regulated activity that unwinds RNA duplexes**. *Cell* 1987, **48**(4):607-613.
2. Rebagliati MR, Melton DA: **Antisense RNA injections in fertilized frog eggs reveal an RNA duplex unwinding activity**. *Cell* 1987, **48**(4):599-605.
3. Nishikura K: **A-to-I editing of coding and non-coding RNAs by ADARs**. *Nature reviews Molecular cell biology* 2016, **17**(2):83-96.
4. Rieder LE, Reenan RA: **The intricate relationship between RNA structure, editing, and splicing**. *Seminars in cell & developmental biology* 2012, **23**(3):281-288.
5. Liu H, Ma CP, Chen YT, Schuyler SC, Chang KP, Tan BC: **Functional Impact of RNA editing and ADARs on regulation of gene expression: perspectives from deep sequencing studies**. *Cell & bioscience* 2014, **4**:44.

6. Deffit SN, Hundley HA: **To edit or not to edit: regulation of ADAR editing specificity and efficiency**. *Wiley interdisciplinary reviews RNA* 2016, **7**(1):113-127.
7. Nigita G, Veneziano D, Ferro A: **A-to-I RNA Editing: Current Knowledge Sources and Computational Approaches with Special Emphasis on Non-Coding RNA Molecules**. *Frontiers in bioengineering and biotechnology* 2015, **3**:37.
8. Daniel C, Lagergren J, Ohman M: **RNA editing of non-coding RNA and its role in gene regulation**. *Biochimie* 2015, **117**:22-27.
9. Slotkin W, Nishikura K: **Adenosine-to-inosine RNA editing and human disease**. *Genome medicine* 2013, **5**(11):105.
10. Tomaselli S, Locatelli F, Gallo A: **The RNA editing enzymes ADARs: mechanism of action and human disease**. *Cell and tissue research* 2014, **356**(3):527-532.
11. Li JB, Church GM: **Deciphering the functions and regulation of brain-enriched A-to-I RNA editing**. *Nature neuroscience* 2013, **16**(11):1518-1522.
12. Sakurai M, Ueda H, Yano T, Okada S, Terajima H, Mitsuyama T, Toyoda A, Fujiyama A, Kawabata H, Suzuki T: **A biochemical landscape of A-to-I RNA editing in the human brain transcriptome**. *Genome research* 2014, **24**(3):522-534.
13. Dominissini D, Moshitch-Moshkovitz S, Amariglio N, Rechavi G: **Adenosine-to-inosine RNA editing meets cancer**. *Carcinogenesis* 2011, **32**(11):1569-1577.
14. Galeano F, Tomaselli S, Locatelli F, Gallo A: **A-to-I RNA editing: the "ADAR" side of human cancer**. *Seminars in cell & developmental biology* 2012, **23**(3):244-250.
15. Paz-Yaacov N, Bazak L, Buchumenski I, Porath HT, Danan-Gotthold M, Knisbacher BA, Eisenberg E, Levanon EY: **Elevated RNA Editing Activity Is a Major Contributor to Transcriptomic Diversity in Tumors**. *Cell reports* 2015, **13**(2):267-276.
16. Han L, Diao L, Yu S, Xu X, Li J, Zhang R, Yang Y, Werner HM, Eterovic AK, Yuan Y *et al*: **The Genomic Landscape and Clinical Relevance of A-to-I RNA Editing in Human Cancers**. *Cancer cell* 2015, **28**(4):515-528.
17. Peng Z, Cheng Y, Tan BC, Kang L, Tian Z, Zhu Y, Zhang W, Liang Y, Hu X, Tan X *et al*: **Comprehensive analysis of RNA-Seq data reveals extensive RNA editing in a human transcriptome**. *Nature biotechnology* 2012, **30**(3):253-260.
18. Park E, Williams B, Wold BJ, Mortazavi A: **RNA editing in the human ENCODE RNA-seq data**. *Genome research* 2012, **22**(9):1626-1633.
19. Bass B, Hundley H, Li JB, Peng Z, Pickrell J, Xiao XG, Yang L: **The difficult calls in RNA editing. Interviewed by H Craig Mak**. *Nature biotechnology* 2012, **30**(12):1207-1209.
20. Ramaswami G, Lin W, Piskol R, Tan MH, Davis C, Li JB: **Accurate identification of human Alu and non-Alu RNA editing sites**. *Nature methods* 2012, **9**(6):579-581.
21. Ramaswami G, Zhang R, Piskol R, Keegan LP, Deng P, O'Connell MA, Li JB: **Identifying RNA editing sites using RNA sequencing data alone**. *Nature methods* 2013, **10**(2):128-132.

22. Bazak L, Haviv A, Barak M, Jacob-Hirsch J, Deng P, Zhang R, Isaacs FJ, Rechavi G, Li JB, Eisenberg E *et al*: **A-to-I RNA editing occurs at over a hundred million genomic sites, located in a majority of human genes**. *Genome research* 2014, **24**(3):365-376.
23. Zhang Q, Xiao X: **Genome sequence-independent identification of RNA editing sites**. *Nature methods* 2015, **12**(4):347-350.
24. Ahn J, Xiao X: **RASER: reads aligner for SNPs and editing sites of RNA**. *Bioinformatics* 2015, **31**(24):3906-3913.
25. Wang Z, Lian J, Li Q, Zhang P, Zhou Y, Zhan X, Zhang G: **RES-Scanner: a software package for genome-wide identification of RNA-editing sites**. *GigaScience* 2016, **5**(1):37.
26. Lee JH, Ang JK, Xiao X: **Analysis and design of RNA sequencing experiments for identifying RNA editing and other single-nucleotide variants**. *Rna* 2013, **19**(6):725-732.
27. Bishop CM: **Pattern Recognition and Machine Learning**: Springer; 2006.
28. Yuan Y, Xu H, Leung RK: **An optimized protocol for generation and analysis of Ion Proton sequencing reads for RNA-Seq**. *BMC genomics* 2016, **17**:403.
29. Dobin A, Davis CA, Schlesinger F, Drenkow J, Zaleski C, Jha S, Batut P, Chaisson M, Gingeras TR: **STAR: ultrafast universal RNA-seq aligner**. *Bioinformatics* 2013, **29**(1):15-21.
30. Kim D, Langmead B, Salzberg SL: **HISAT: a fast spliced aligner with low memory requirements**. *Nat Methods* 2015, **12**(4):357-360.
31. Li Q, Wang Z, Lian J, Schiott M, Jin L, Zhang P, Zhang Y, Nygaard S, Peng Z, Zhou Y *et al*: **Caste-specific RNA editomes in the leaf-cutting ant Acromyrmex echinatior**. *Nature communications* 2014, **5**:4943.
32. LeCun Y, Bengio Y, Hinton G: **Deep learning**. *Nature* 2015, **521**(7553):436-444.
33. Xiong HY, Alipanahi B, Lee LJ, Bretschneider H, Merico D, Yuen RK, Hua Y, Gueroussov S, Najafabadi HS, Hughes TR *et al*: **RNA splicing. The human splicing code reveals new insights into the genetic determinants of disease**. *Science* 2015, **347**(6218):1254806.
34. Xiong H, Liu D, Li Q, Lei M, Xu L, Wu L, Wang Z, Ren S, Li W, Xia M, Lu L, Lu H, Hou Y, Zhu S, Liu X, Sun Y, Wang J, Yang H, Wu K, Xu X, Lee LJ: Supporting data for "RED-ML: a novel, effective RNA editing detection method based on machine learning" *GigaScience* Database. 2017. http://dx.doi.org/10.5524/100275
35. RED-ML project homepage. https://github.com/BGIRED/RED-ML. Accessed 27 December, 2016.

Figure Captions

Fig. 1 Flow charts of our RED-ML pipeline: (a) overview of the entire pipeline; (b) schematic of the ML component in RED-ML.

Fig. 2 Evaluating RED-ML on the YH dataset. (a) & (b) ROC and precision-recall curves on the test set when building the LR classifier. Both curves were plotted to show a more

comprehensive picture of RED-ML performance on this biased dataset, where the number of negative examples is about twice of the positive ones, and they are obtained by varying the detection threshold in small steps. (c) The effect of varying the detection threshold: the Ion Proton validation rate increases monotonically as more stringent classification thresholds are chosen. (d)-(f) Adjusting the detection threshold to compare RED-ML with the methods of Peng et al, Ramaswami et al and RES-scanner: the thresholds used are 0.96, 0.5 and 0.68, respectively.

Fig. 3 Evaluating RED-ML on two prostate tumor samples (CH24T and CH62T) and a Hela sample: (a) number of detected RNA editing sites and (b) Ion Proton validate rates by RED-ML (using the default detection threshold of 0.5) and RES-scanner in the three samples; (c) MS validation of some RNA editing sites detected by RED-ML and RES-scanner in CH24T and CH62T; (d) the effect of varying the detection threshold in CH24T.

Fig. 4 Further analysis of the RED-ML detected sites. (a)-(c): The overlap of detected sites with two curated RNA editing databases (DARNED and RADAR) in CH24T, CH62T and Hela samples were shown as Venn diagrams. (d) Ion Proton validation rates for different classes of sites (defined in the main text) in the three samples. The number of validated sites in each class is also indicated on the top of each bar. (e) The percentage of genomic variants in detected RNA editing sites as quantified by matching DNA sequencing data.

Click here to download Figure Figure1.pdf

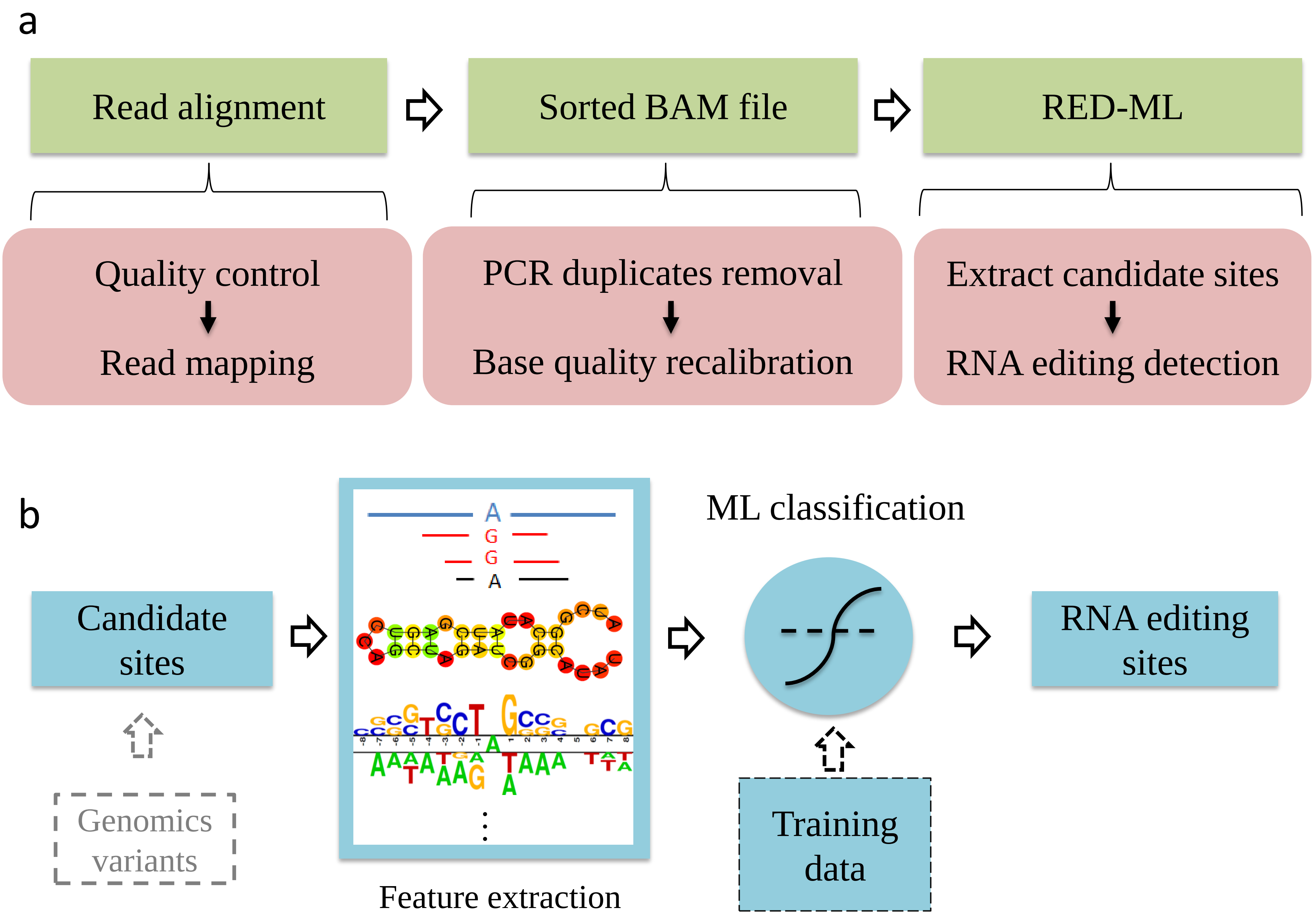

Figure 2

Click here to download Figure Figure2.pdf

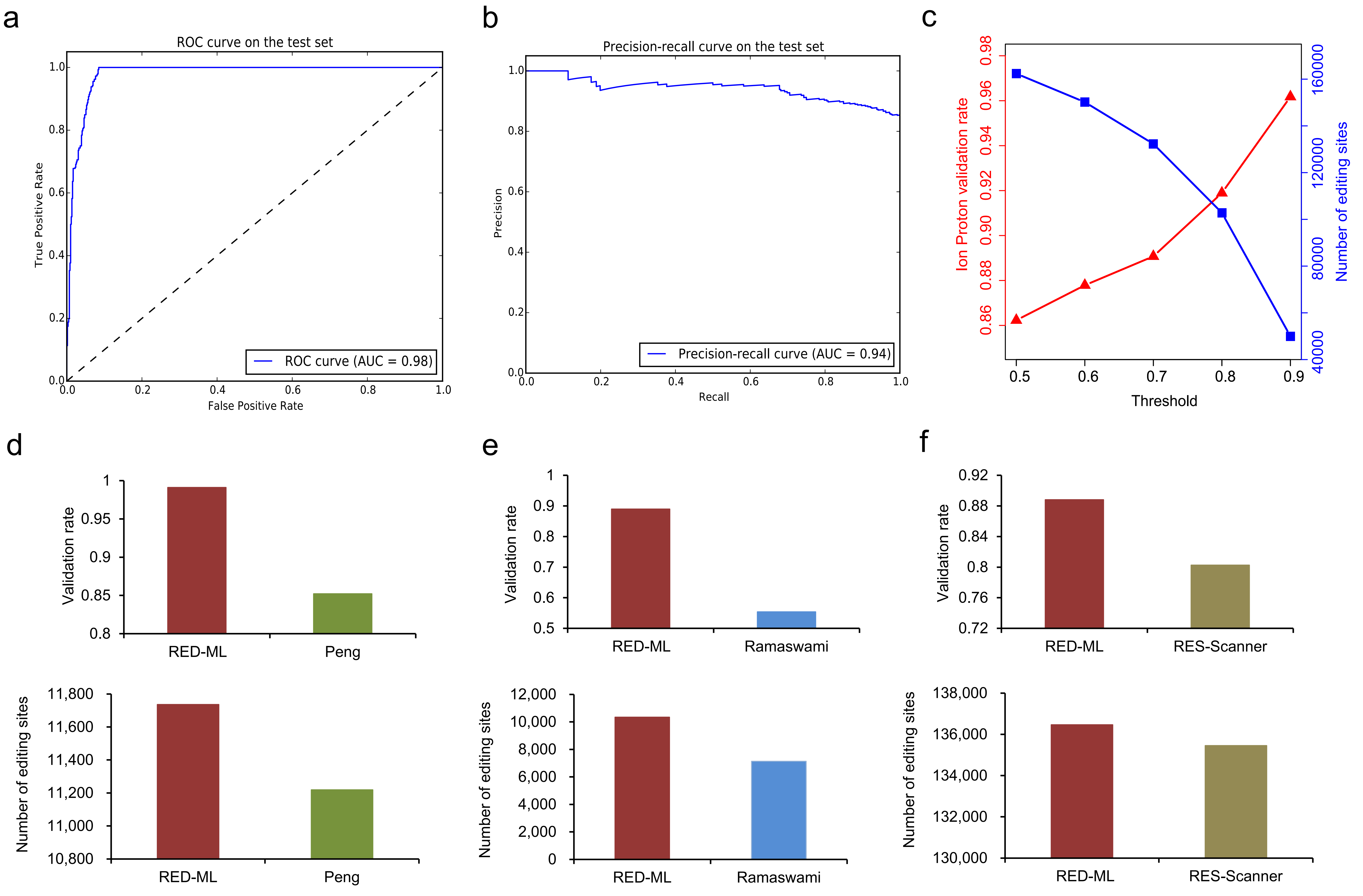

Figure 3

Click here to download Figure Figure3.pdf

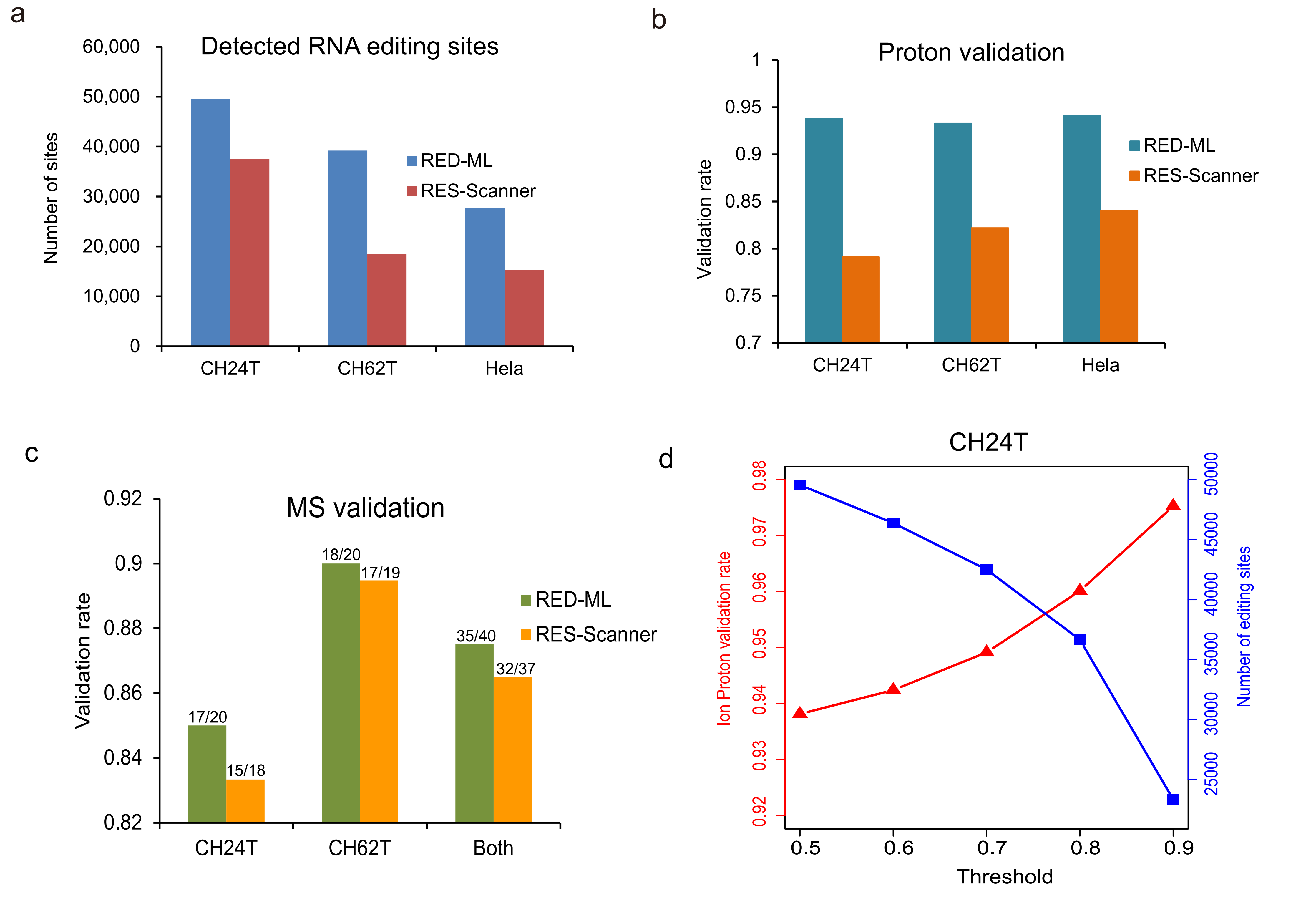

Figure 4

Click here to download Figure Figure4.pdf

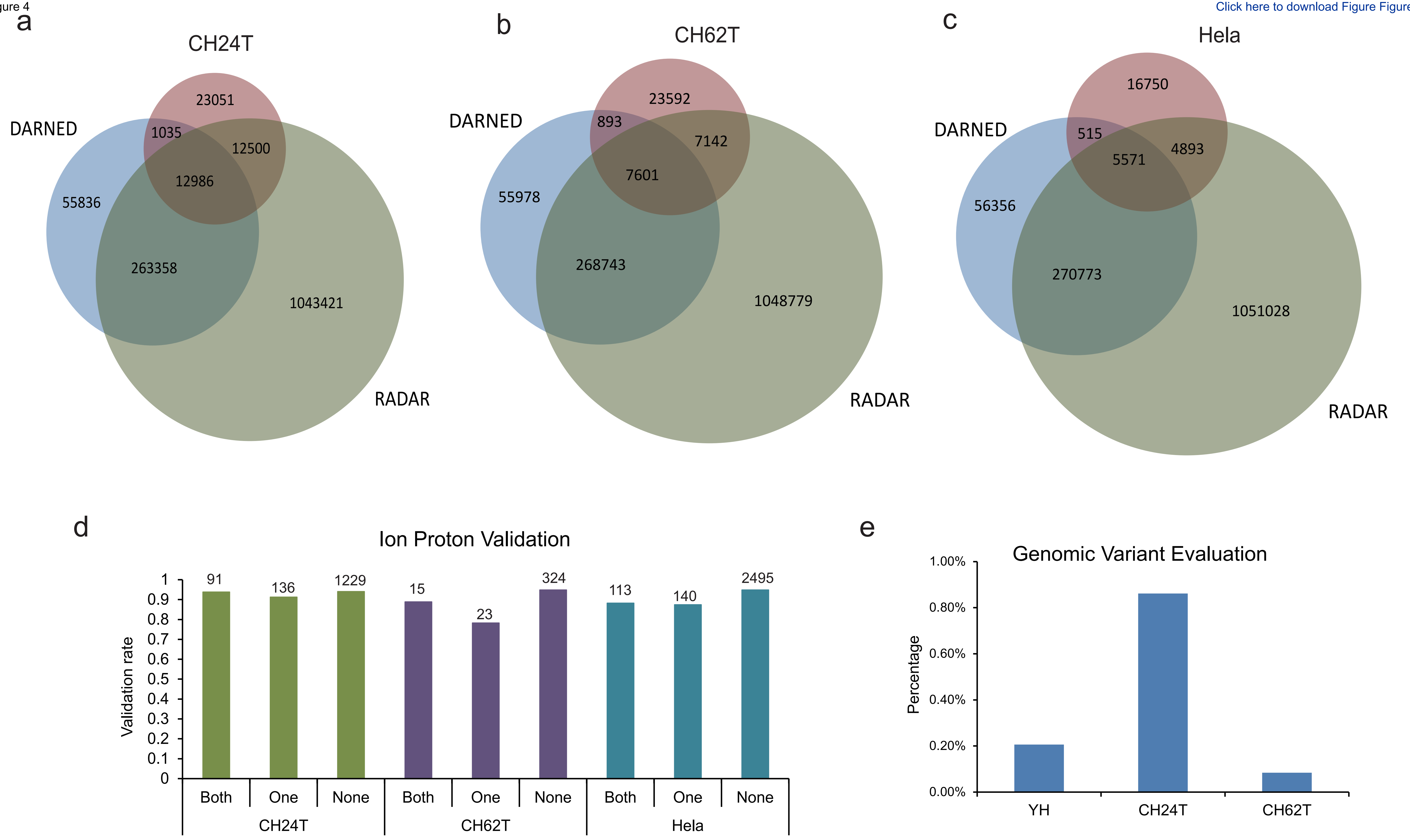

Click here to access/download
Supplementary Material
SM.docx

Figure S1

Click here to access/download
Supplementary Material
FigureS1.pdf

Figure S2

Click here to access/download
Supplementary Material
FigureS2.pdf

Figure S3

Click here to access/download
Supplementary Material
FigureS3.pdf

Figure S4

Click here to access/download
Supplementary Material
FigureS4.pdf

Figure S5

Click here to access/download
Supplementary Material
FigureS5.pdf

Figure S6

Click here to access/download
Supplementary Material
FigureS6.pdf

Figure S7

Click here to access/download
Supplementary Material
FigureS7.pdf

Figure S8

Click here to access/download
Supplementary Material
FigureS8.pdf

Table S1-S10

Click here to access/download
Supplementary Material
TableS1-10.xlsx

Table S11

Click here to access/download

Supplementary Material

TableS11.xlsx

 Click here to download Personal Cover RED-ML-Response.docx

GIGA-D-16-00054
RED-ML: a novel, universal RNA editing detection method based on machine learning
GigaScience

Dear Dr. Zauner,

Thank you for giving us the opportunity to resubmit our manuscript! Based on the reviewers' comments and your suggestions, and also in light of our recently published RES-scanner paper (Wang et al, *GigaScience* 2016), we have made substantial changes to our original submission. This has resulted in a much improved manuscript and we believe that we have fully addressed all concerns from the reviewers. The major changes are summarized below:

1. Since our previous in-house hard filter is an adapted and optimized version of RES-scanner on the YH dataset, we now simply refer to it as RES-scanner when describing our model (with detailed differences provided in the SM). We feel that this has added transparency and consistency to our Method section.

2. Based on the experience gained from trying different alignment strategies in RES-scanner, we now recommend BWA as the default alignment tool. We have updated all figures based on this improved alignment strategy and it has resulted in considerably better performance than before. RED-ML can detect more RNA editing sites with similarly high validation rates in all samples comparing to the previous submission. Moreover, the performance advantage of RED-ML with respect to other hard filtering based methods, including RES-scanner, also becomes more evident. RED-ML can still work well with TopHat2 and we have put the previous results in SM. We have also added more thorough discussion on different alignment strategies in the Discussion section.

3. In terms of comparing with other methods, we have made full comparisons with RES-scanner in all cases and shown that RED-ML has much improved accuracy and speed. Since it has already been demonstrated that RES-scanner is superior to other existing RED methods, we believe this fully addressed the request of adding more comparisons from the reviewers.

4. The second reviewer suggested that we could use simulation to stress test our model and see when it would fail. Although we have carried out various simulations when building our model, we didn't include them in the manuscript since they didn't help much in evaluating the real world performance of RED-ML in our opinion (details in the response to reviewers below). However, we do agree with the reviewer that it would be helpful to stress test our method and show its limitation. In order to do so, we have added the analyses to run RED-ML with very different alignment strategies and on the ant RNA-seq data, a species very different from human, and included these results in the Discussion section.

5. We have also made various improvements on the RED-ML software and it is fully open source now. Details are in SM and the GitHub page.

There are also many minor changes to improve the quality of the manuscript, such as precise reference to test data, better explanations of our ML philosophy and validation procedure, detailed in our point-to-point response to the reviewers below and in the revised manuscript. In summary, we are highly confident that the revised manuscript meets the requirement of being published in GigaScience. We are looking forward to a speedy response from you as last time. Thank you very much!

Yours sincerely,

Leo

Leo J Lee, PhD
BGI-shenzhen & University of Toronto
Email: ljlee@psi.toronto.edu
Phone: +1 4168865650, +86 13148700528

Below is a point-to-point response to the reviewers' comments.

Reviewer reports:

Reviewer #1, Anton Feenstra: The authors present a manuscript on a machine learning approach to detecting rna editing events in (NGS) sequencing data. The manuscript is concise, generally clear, and the results appear convincing.

We thank the positive comments from this reviewer!

I have, however, a few concerns.

My main concern is the very high accuracy the authors report for their method. An AUC of around 95% in ROC or P/R plot is exceptional; achieving this high accuracy suggests the problem is actually fairly simple. It is therefore surprising that this result was not reached earlier in the roughly 15 years since the start of studying rna editing events in (NGS) sequencing data. This point warrants a deeper discussion than is currently presented in the manuscript.

We thank the reviewer for pointing this out! Indeed, the very high accuracy shown by the ROC and P-R plots does not represent the true performance of RED-ML. It only shows the test error when building our LR classifier on the 4,400 data points, and confirms the adequacy of adopting a relative simple machine learning technique for this task. We have clarified this in our revised manuscript. That's also why we carried out independent RNA-seq experiments with Ion Proton sequencing validation to evaluate the real world performance of RED-ML on CH24T, CH62T and Hela and show that it can generalize well beyond the specific dataset used to build the model.

In addition, to support this discussion, ROC and P-R plots and corresponding AUC for the other methods (Peng, Ramaswami, and their 'in-house hard filtering') should also be presented in the manuscript.

One main advantage of RED-ML is that it also produces a confidence score when detecting RNA editing sites, which can be varied to obtain ROC and P-R plots. Hard filtering based methods don't have such a desirable property. Therefore, there are no easy ways to obtain full ROC and P-R plots for them.

Similarly, validation results as presented in Fig 3 for the new method, should also be presented for the other three methods.

We have added full comparison to our recently published hard filtering based method RES-scanner to show the advantage of RED-ML. Since RES-scanner has already been

demonstrated to be superior to other hard filtering based RED methods (Wang et al, GigaScience 2016), we believe there is no need to compare with them again.

Lesser concerns, approximately in decreasing order of importance:

- in Results, 'unseen test data' is mentioned in relation to Fig 2. It should be specified which test set this refers to specifically.

- *The unseen test data is 20% of the 4,400 data points that was randomly sampled and not used in training. We have clarified this in our revised manuscript.*

- the latent variable used for producing the ROC and P-R plots (e.g. in Fig 2) is not defined in methods or results, nor in the captions.

- *We are sorry about this oversight! The latent variable is the confidence score, and this has been added to the figure caption.*

- in Discussion, the new method is described as 'user-friendly and easy to use'. I believe this statement is insufficiently substantiated in the results presented.

- *We thank the reviewer for the critical view! What we meant was that comparing to most previous RED methods, we provided an all-in-one software package that could be conveniently invoked as a single command from the command line. We have made this clear and also tuned down this statement a bit in the revised manuscript.*

- the first paragraph of Methods is somewhat opaque:
  In the revised manuscript, we have omitted the exact details of BAM file processing since it is not the focus of RED-ML, and the tool can also accept any sorted BAM files provided by a user. Exact details of our recommended steps are provided in SM, and there are further discussions about different read alignment strategies at the end of the revised manuscript.

- what exactly should be done to obtain 'a post-processed and sorted BAM file' (a suitable literature reference might suffice)?
  As stated previously, this has been removed from the revised manuscript and details of the recommended steps to process BAM files are provided in SM. Briefly, for Tophat2, Picard was used to sort BAM files and to remove PCR duplicate reads, then base quality score recalibration was carried out by GATK. For BWA, SAMtools was used to sort BAM files and to remove PCR duplicate reads.

- the RASER tool is highlighted as having special properties relating to SNP and RNA editing detection. It should be explained what this tool exels at, and how that is achieved.

- *We have removed this sentence in our revised manuscript to avoid confusion. This is a claim made by the author of RASER, not our own. Some discussions on different alignment tools are provided in the Discussion section.*

- the reference to 'best practices' is unclear, and is not helped by stating it 'includes' some steps, with a literature reference. Are (all) other steps of 'best practices' included in this reference? This should be made clear.
- *This has been removed in our revised manuscript while exact details are briefly mentioned above and also provided in SM.*

- in the second paragraph of Methods, the relevance of Alu regions in the context of RED should be explained. Also, it should be explained why 'hard decisions' cannot be made on the 'third class' of features (which include the Alu regions).
- *We wish to incorporate the knowledge that RNA editing happens preferably in Alu regions, so we include this feature in RED-ML. However, such a feature alone (and others in the "third class") cannot be used to directly filter out non RNA editing sites, which is what we meant by "it is inappropriate to make hard decisions based on them". Although it is possible to design different hard filters based on whether a potential RNA editing site resides in Alu repeat regions or not, as done by Ramaswami et al, this is clearly a very inefficient approach. The number of hard filter classes would grow exponentially as the number of third class features grows. Therefore, making use of them under the ML framework is a much more principled approach.*

- in the 'Building a ML classifier' section, the construction of the training set is not very clear. In particular, it should be explained why the additional 141 positives (by Peng & Ramaswami, but not their method, and validated) are needed, particularly since it only increases the positive set by 10%. It should also be explained why sites that do not pass the hard filtering criteria are a good component of the negative set; these are probably the most obvious ones that would not need to be predicted anyway.
- *Besides the overview provided in the main text, full details of constructing the training set are provided in SM. Our ML based method is designed to be an improvement of our previous hard filtering based method. It needs to first learn what the hard filtering method can already achieve, which is why some samples of data points not passing the hard filtering criteria should be included in the negative set. Moreover, the additional 141 data points are the true editing sites that the hard filtering method was not able to detect, and it is important to include them (although the size is relatively small) so that RED-ML can learn to perform better than the hard filtering method. Likewise, it is also important to include the 375 data points that has been misclassified by our hard filter in the negative set.*

- the 'YH' label for the dataset from ref 17 is not introduced, which is somewhat confusing. Was this dataset labeled such by the authors of 17?
- *We are sorry about the oversight! The YH dataset is the same as the male Han Chinese individual RNA-seq and DNA-seq data in the reference (it has been*

traditionally called the YH dataset within BGI). We have made this clear in the revised manuscript.

- in the caption of Fig 2 the phrase 'on a test fold' does not make sense to me.
- *This is the same as the 20% of 4,400 data points used as the test data. We have clarified this in the main text as well as the figure caption in the revised manuscript.*

Typos:

- "Since sites predicted by Peng et al [17] tends..." --> please correct to "... tend ..." (remove plural 's')
- *We thank the careful reviewer for pointing this out and we have corrected it.*

Reviewer #2, Fabrizio Costa: The authors present a tool for RNA editing events detection. The tool is based on a logisitc regression classifier over 28 features and trained over 1300 positives and 3000 negative examples.

The work would benefit from a clearer (if not formal) definition of the task at hand and a better introduction to defend the importance of the problem.

The task we are trying to accomplish is genome-wide RNA editing detection from RNA-seq. We have added a sentence to clarify this in the last paragraph of Introduction. The importance of this task can be summarized as: 1) The biological importance of RNA editing has been well established in the literature; 2) RNA-seq is the first, and so far the only, experimental technique to profile RNA editing on a genome-wide scale; 3) Detecting RNA editing sites from RNA-seq is a very challenging computational problem (e.g., as thoroughly discussed in Bass et al, Nature Biotechnolgy 2012) and good tools are seriously lacking. We feel that we have adequately supported the above three statements in our Introduction. If the reviewer has specific concerns, we will be glad to address them further.

The definition of the validation procedure that uses proton sequencing and the agreement with a competitive tool needs to be better detailed and justified.

We thank the reviewer for pointing this out. As Ion Proton sequencing may not be familiar to many readers, in the revised manuscript we have added the following sentences to briefly introduce it and justify our validation method, besides adding an appropriate reference.
"Although both Ion Proton sequencing and Illumima Hiseq are referred to as second generation sequencing platforms, they differ in many key aspects, including the underlying chemistry, base calling method as well as read alignment strategies. We took advantage of these differences to perform independent, high-throughput validation of the RNA-editing sites detected by Hiseq."
We have also provided more details in SM for further clarification. As to competitive tools, we guess the reviewer meant competitive, experimental validation methods. Since

previous validation methods in the literature are all of low throughput, such as Sanger sequencing and mass spectrometry, we don't have a competitive method to properly compare against. In fact, the design of such a high throughput validation procedure is an important novel contribution of this work.

The work would benefit from having a section devoted to studying an artificial case where the modeling hypothesis could be manipulated (e.g. the frequency of the editing events) to show under which circumstances the method would start failing or not being reliable any more.

We thank the reviewer for the suggestion! We have indeed carried out various simulations when building our model, such as varying the editing frequency and read coverage. However, we decided not to include them in the manuscript since these simulations are not able to faithfully mimic the effect of RNA editing on RNA-seq. In fact, there are no well accepted methods to simulate RNA editing due to the lack of understanding of this process, unlike gene expression analysis for example, and that is why no RNA editing detection methods have been tested on meaningful simulations before. As a result, our own simulations didn't contribute much to evaluating the real world performance of RED-ML either. They are most useful as debugging tools instead. But we do agree with the reviewer that it is helpful to see when the method starts to fail, and we have carried out two experiments to stress test our method. First we tried a very different alignment strategy based on STAR, which has not been used when building the training set. On the CH24T dataset, a total of 246,879 RNA editing sites were detected, but the validation rate is very low (0.34), so there are probably many false positives. Second although our method was built to do RED for human, we tested it on RNA-seq data from a very different species — ant (data from Li et al, Nature Communications 2014). A total of 15,354 RNA editing sites were identified, but the proportion of A-to-I editing was only 0.605. These experiments demonstrate that RED-ML don't work very well in situations that are vastly different from the training set. These results are also further discussed in the Discussion section of the revised manuscript.

The definition of the test set is unclear and hence it is hard to assess how representative the performance estimate really is.

We thank the reviewer for this critical comment and we have made efforts to clarify the test data used in our revised manuscript. Roughly speaking, there are two types of test data used in our work. The first is the test data defined under the classical ML framework. In our revised manuscript, we have made it clear that it is 880 (20% of 4,400) randomly sampled data points that are not used in training. The very good results shown in Fig. 1a&1b are mostly aimed to justify the use of a relatively simple LR classifier after careful feature engineering. The second and more important type of test data comes from independent RNA-seq and validation experiments on CH24, CH64 and Hela. We believe these data sets can give realistic estimates on the performance of RED-ML.

The work would benefit from better detailing the features used. Moreover it would be informative for the readers to have an assessment of the features quality (i.e. via a feature selection procedure).

We have provided details of all features in Table S4. To give readers more insights into our feature set, we added the feature importance analysis as shown in Fig. S3 and Table S4. This is achieved by first normalizing the magnitude of each feature to be no more than 1 in the training set and then comparing the absolute values of the weights for each feature. Feature selection is not so relevant here since we only have 28 manually designed features in total and overfitting has been carefully controlled for.

The software although available on GitHub is not open as it includes some executables without their respective source code.

Thanks for the suggestion! We have now made our software completely open source on GitHub.
