# Supplementary figures and images for: RED-ML: a novel, effective RNA editing detection method based on machine learning
Source: Gigascience. 2017 Mar 2;6(5):1–8. doi: 10.1093/gigascience/gix012 (PMC5467039; doi:10.1093/gigascience/gix012)

# Ion Proton Validation

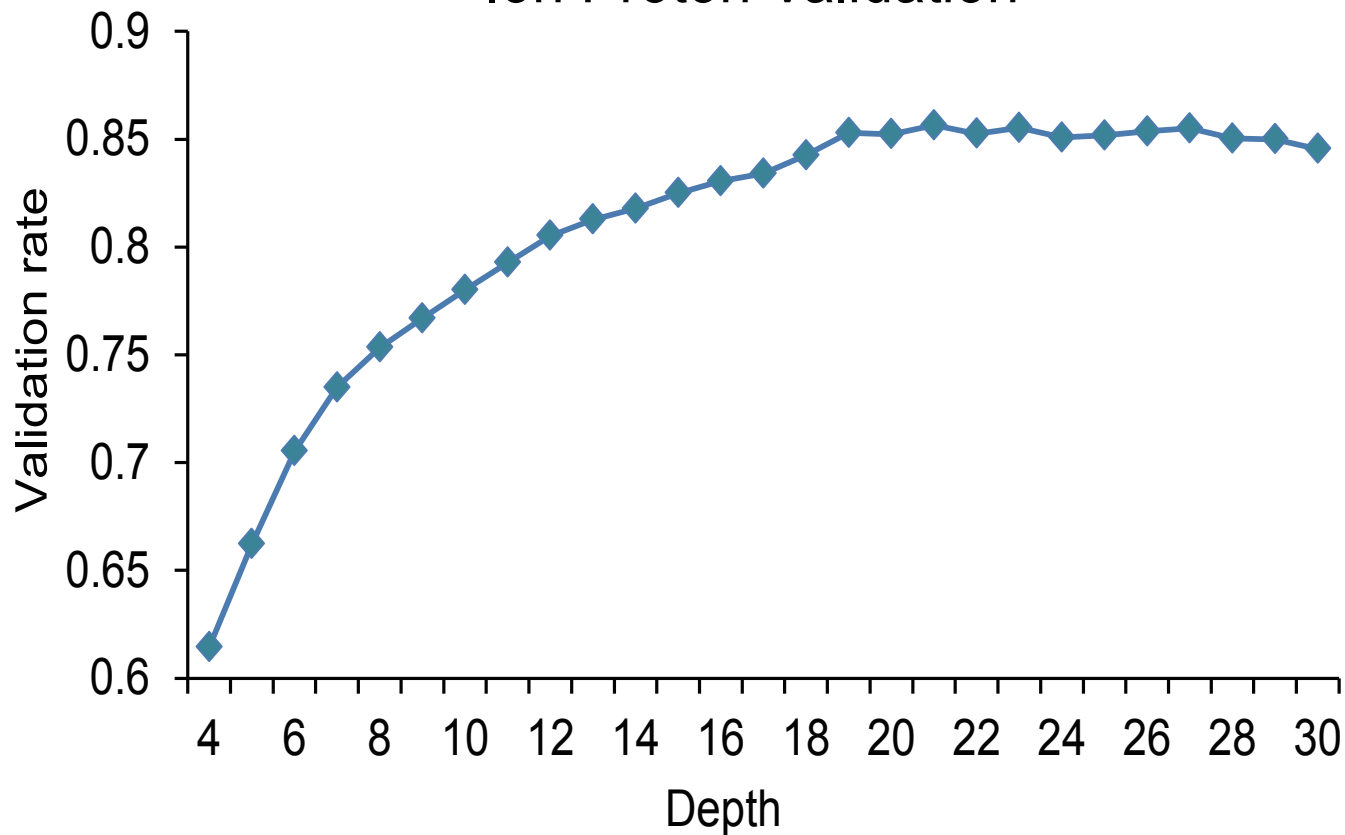

Supplement: FigureS1.pdf [file gix012_FigureS1.pdf]

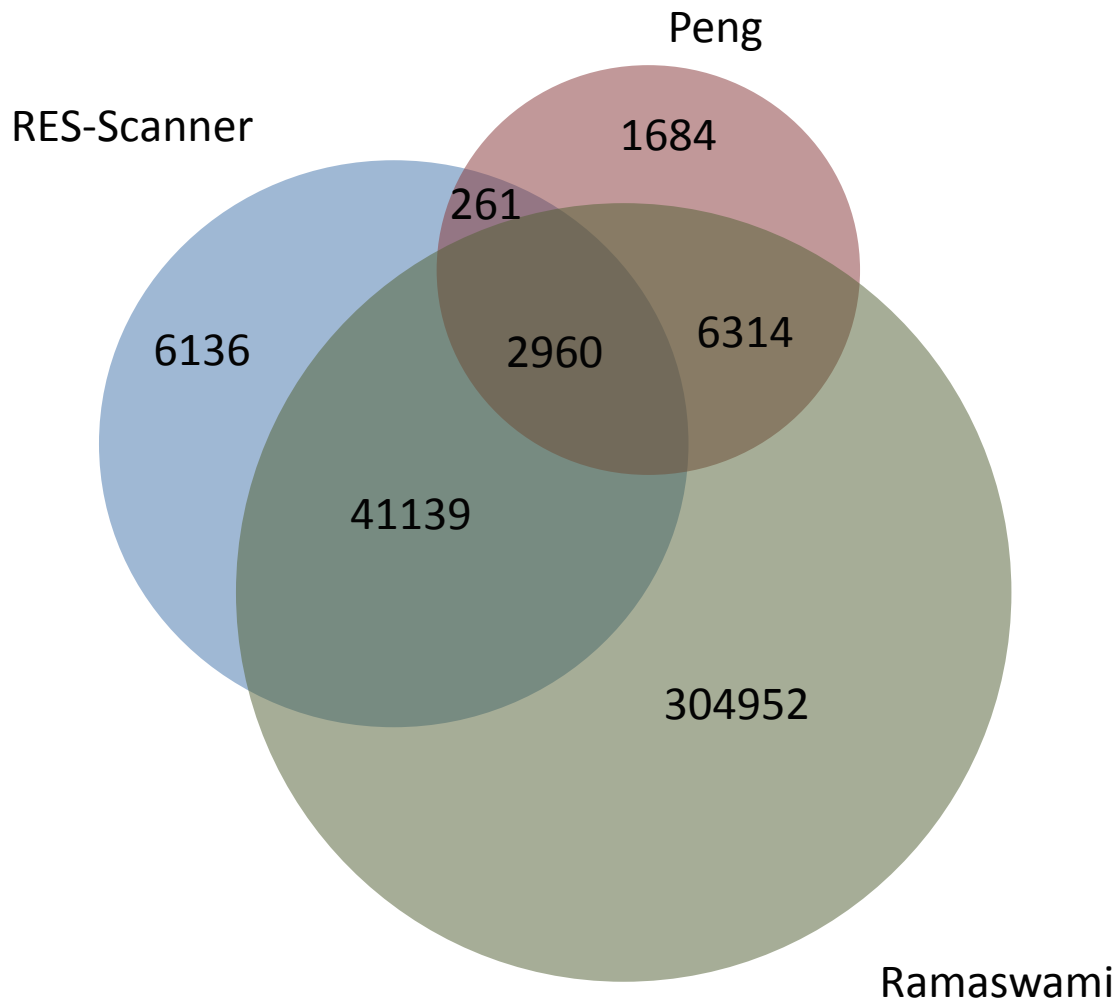

Supplement: FigureS2.pdf [file gix012_FigureS2.pdf]

Features Importance

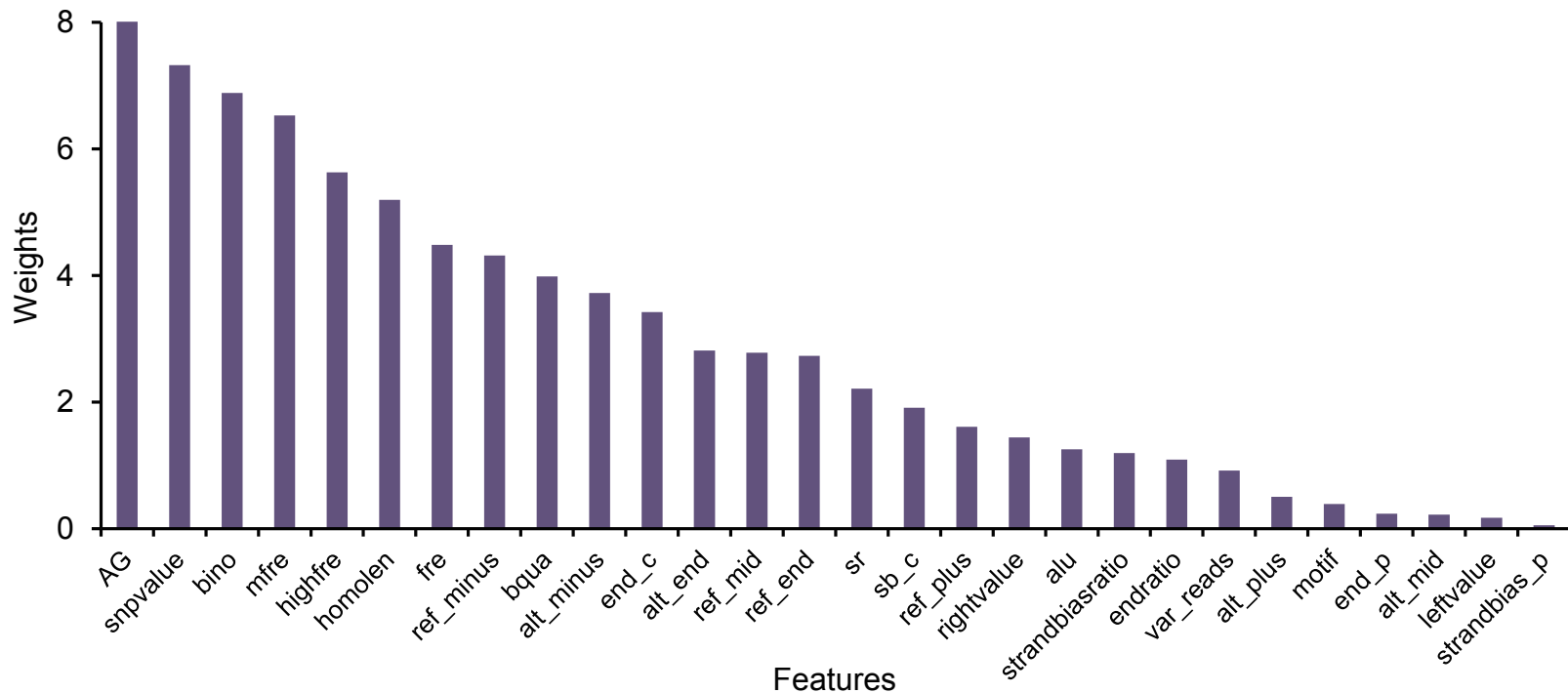

Supplement: FigureS3.pdf [file gix012_FigureS3.pdf]

a

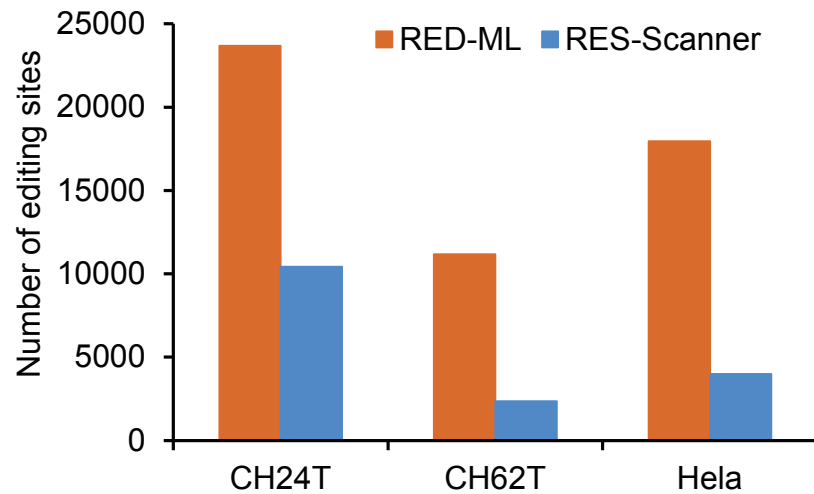

b

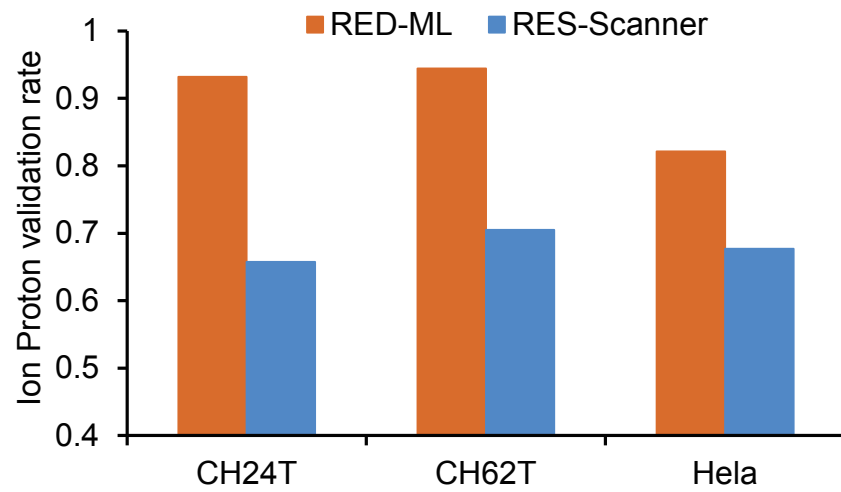

Supplement: FigureS4.pdf [file gix012_FigureS4.pdf]

a

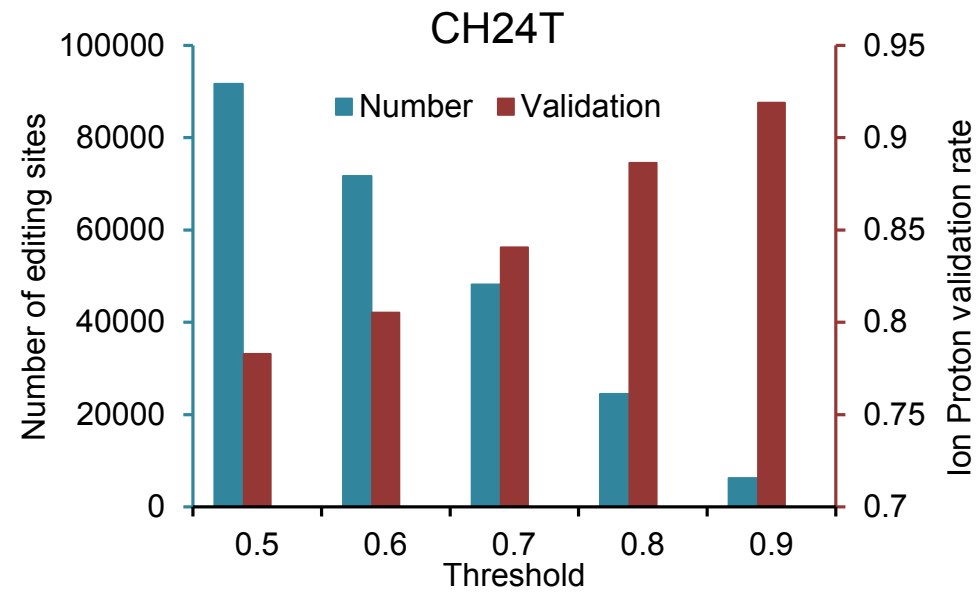

b

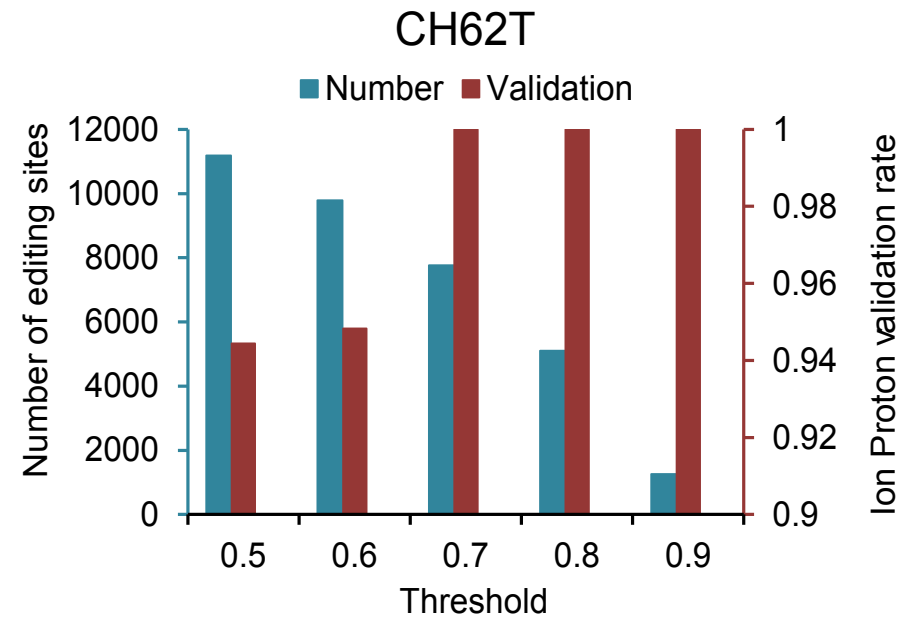

c

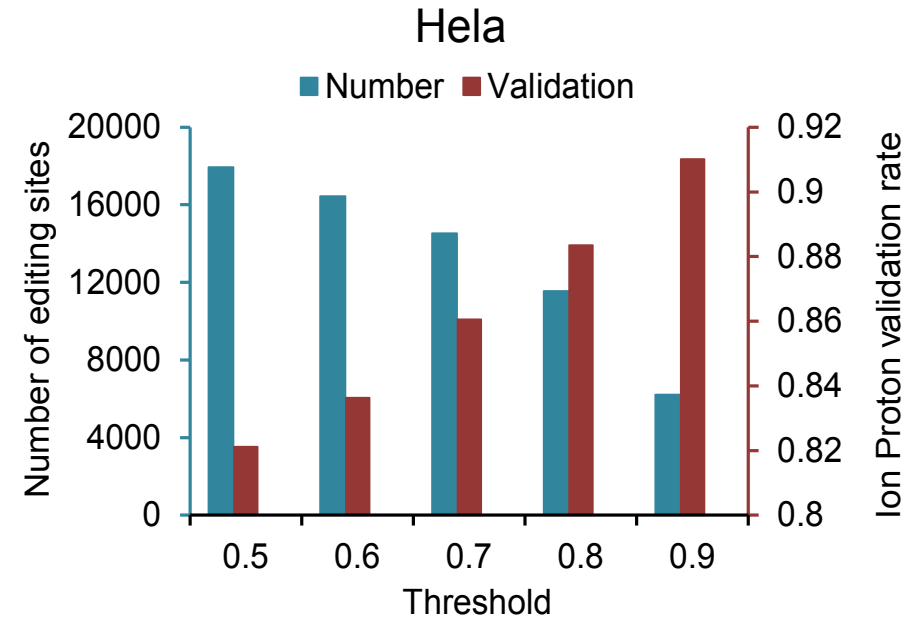

Supplement: FigureS5.pdf [file gix012_FigureS5.pdf]

a

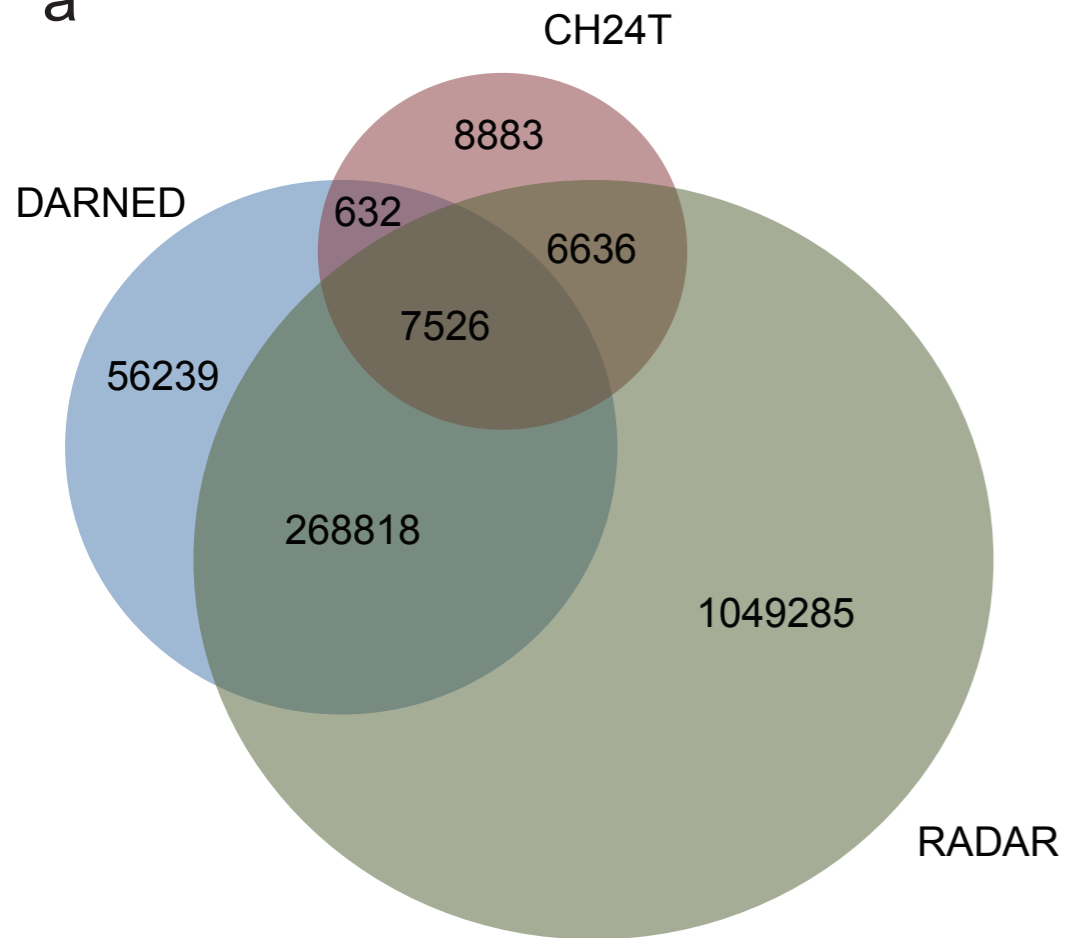

b

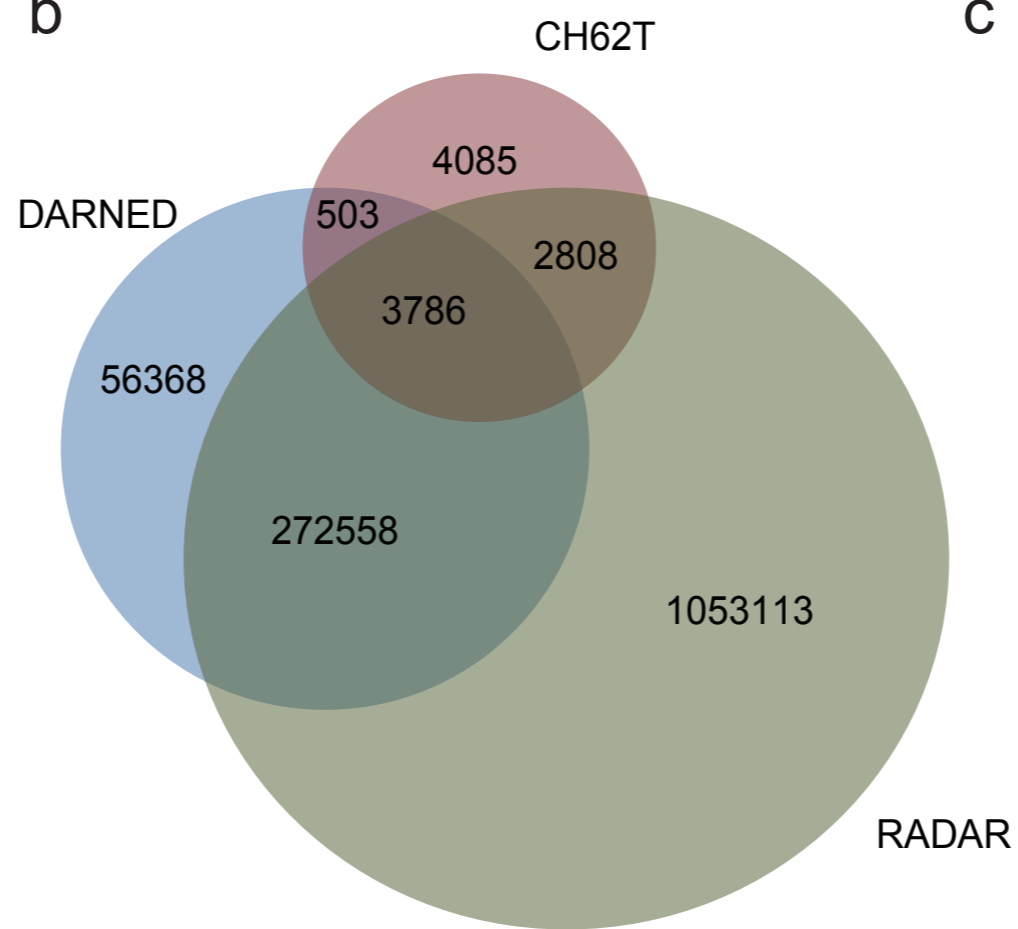

c

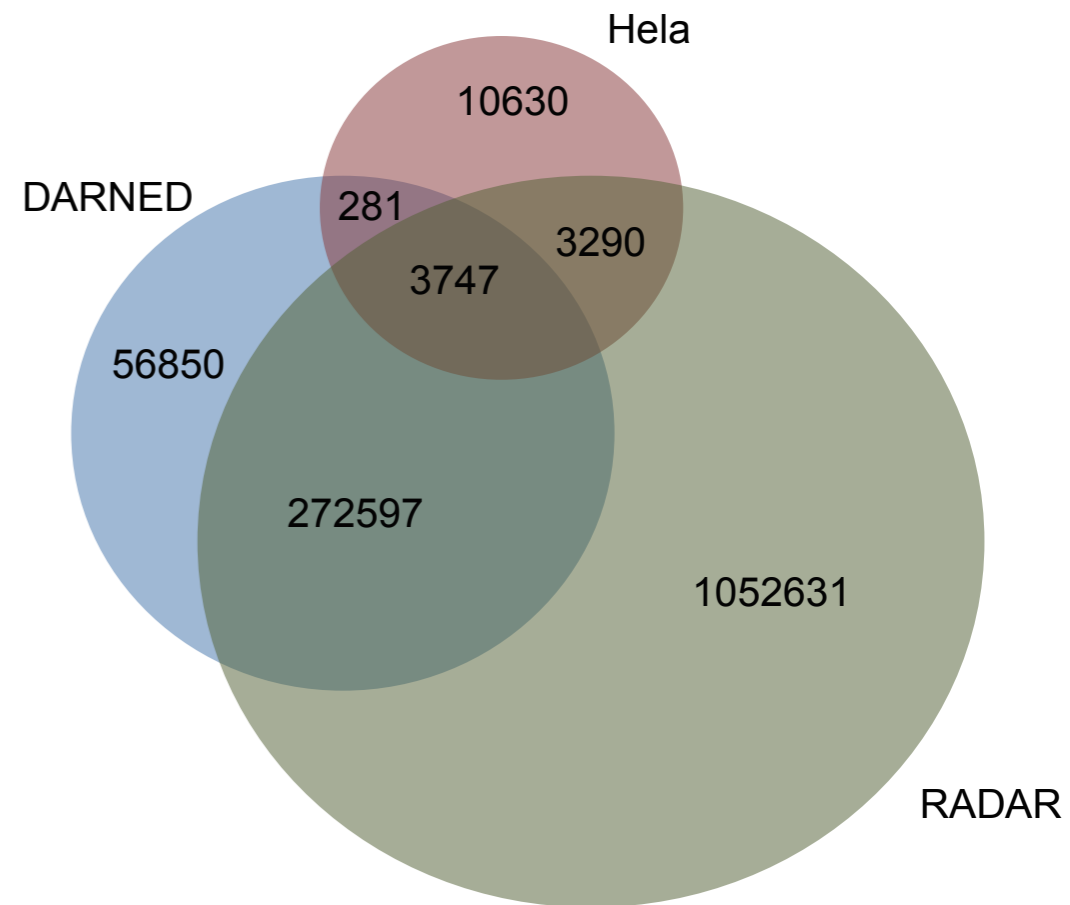

Supplement: FigureS6.pdf [file gix012_FigureS6.pdf]

# Ion Proton Validation

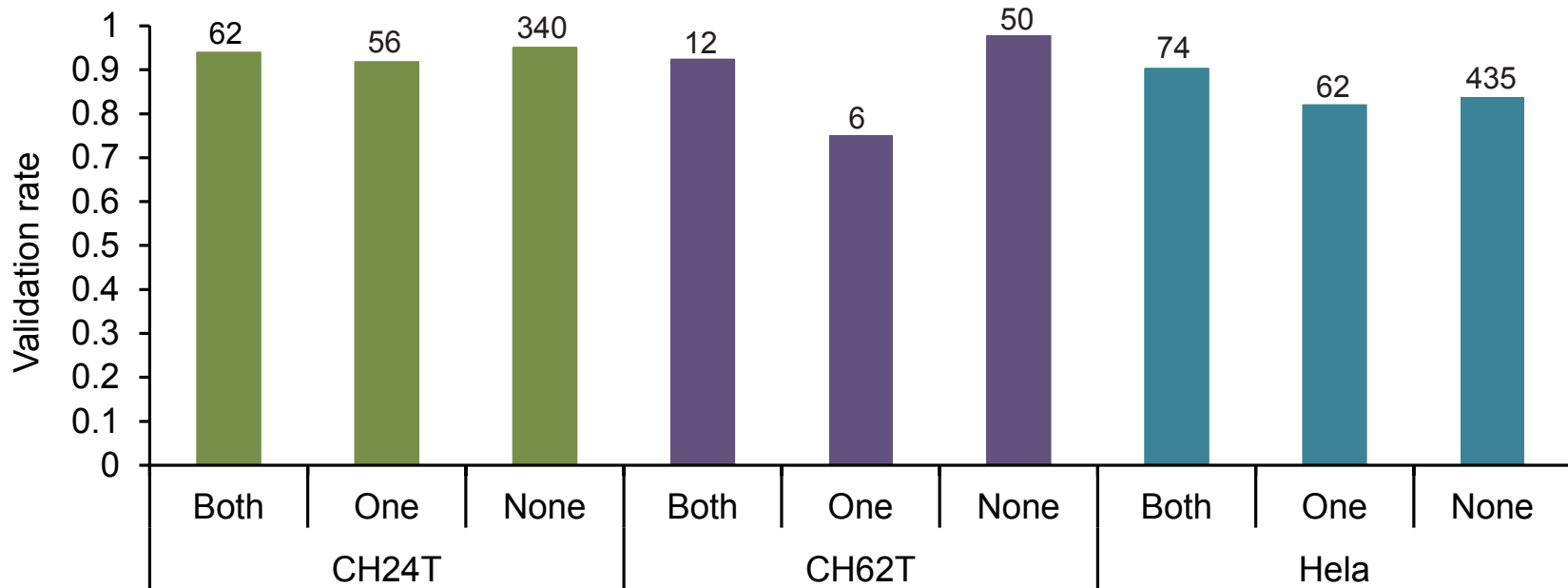

Supplement: FigureS7.pdf [file gix012_FigureS7.pdf]

# Genomic Variant Evaluation

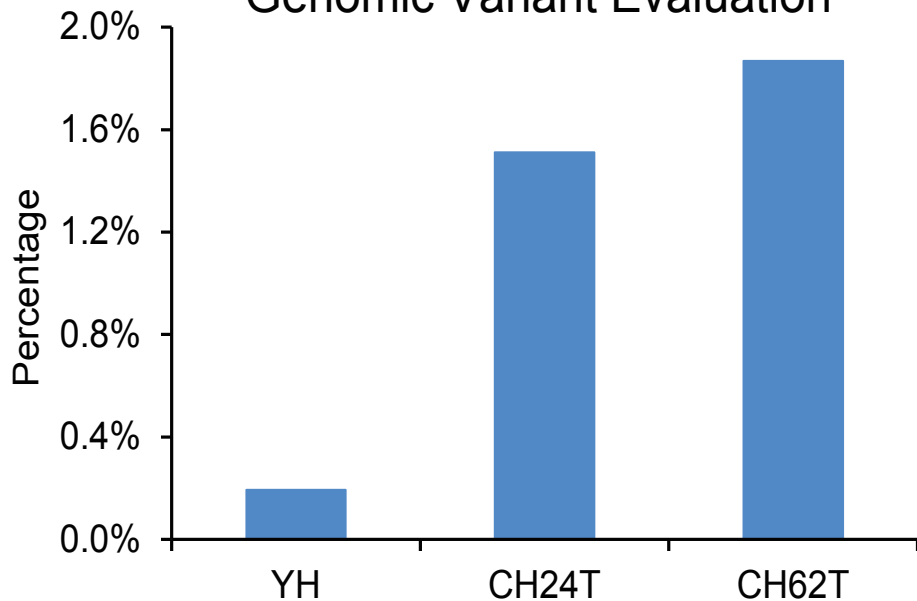

Supplement: FigureS8.pdf [file gix012_FigureS8.pdf]
