# Supplementary material for: RED-ML: a novel, effective RNA editing detection method based on machine learning
Source: Gigascience. 2017 Mar 2;6(5):1–8. doi: 10.1093/gigascience/gix012 (PMC5467039; doi:10.1093/gigascience/gix012)
Supplement: Reviewer_1_Report_(Original_Submission).pdf [file gix012_Reviewer_1_Report_(Original_Submission).pdf]

Reviewer Report

Title: "RED-ML: a novel, effective RNA editing detection method based on machine learning"

Version: Original Submission **Date:** 12/15/2016

Reviewer name: Anton Feenstra

Reviewer Comments to Author:

I am fully satisfied with the way in which the authors have incorporated my comments into their revised manuscript.

Level of Interest

Please indicate how interesting you found the manuscript: An article of importance in its field

Quality of Written English

Please indicate the quality of language in the manuscript: Acceptable

Declaration of Competing Interests

Please complete a declaration of competing interests, considering the following questions:

- Have you in the past five years received reimbursements, fees, funding, or salary from an organisation that may in any way gain or lose financially from the publication of this manuscript, either now or in the future?
- Do you hold any stocks or shares in an organisation that may in any way gain or lose financially from the publication of this manuscript, either now or in the future?
- Do you hold or are you currently applying for any patents relating to the content of the manuscript?
- Have you received reimbursements, fees, funding, or salary from an organization that holds or has applied for patents relating to the content of the manuscript?
- Do you have any other financial competing interests?
- Do you have any non-financial competing interests in relation to this paper?

If you can answer no to all of the above, write 'I declare that I have no competing interests' below. If your reply is yes to any, please give details below.

I declare that I have no competing interests.

I agree to the open peer review policy of the journal. I understand that my name will be included on my report to the authors and, if the manuscript is accepted for publication, my named report including any attachments I upload will be posted on the website along with the authors' responses. I agree for my report to be made available under an Open Access Creative Commons CC-BY license (http://creativecommons.org/licenses/by/4.0/). I understand that any comments which I do not wish to be included in my named report can be included as confidential comments to the editors, which will not be published.

I agree to the open peer review policy of the journal
